# Electrostatic Interaction of Enzyme-Mediated Poly(gallic acid) with Monosodium Urate Crystals Attenuates Inflammasome Activation and Oxidative Stress

**DOI:** 10.3390/polym18151828

**Published:** 2026-07-26

**Authors:** Iris N. Serratos, Luis Angel Carrasco-Sánchez, Karina Martínez-Flores, Roberto Sánchez-Sánchez, Valentín Martínez-López, Ambar López-Macay, Jesús Cervantes-Meneses, Alberto Carlos-Martínez, Rafael Guillermo Suárez-Nájera, Alfredo Jiménez-Mondragón, Ingrid Salgado-Gutiérrez, Carmen G. Hernández-Valencia, Mónica Olvera-Barranco, Janitzia Vázquez-Mellado, Israel Alfonso Núñez-Tapia, Miquel Gimeno, Javier Fernández-Torres, Yessica Zamudio-Cuevas

**Affiliations:** 1 Departamento de Química, Universidad Autónoma Metropolitana, Avenida Ferrocarril San Rafael Atlixco, No. 186. Col. Leyes de Reforma 1^a^ Sección, Alcaldía Iztapalapa, Ciudad de México 09310, Mexico; insa@xanum.uam.mx (I.N.S.);; 2 Departamento de Ciencias Biológicas y de la Salud, Universidad Autónoma Metropolitana, Avenida Ferrocarril San Rafael Atlixco, No. 186. Col. Leyes de Reforma 1^a^ Sección, Alcaldía Iztapalapa, Ciudad de México 09310, Mexico; cbs2183020593@izt.uam.mx; 3 Laboratorio de Líquido Sinovial, Instituto Nacional de Rehabilitación Luis Guillermo Ibarra Ibarra, Calzada México-Xochimilco, No. 289. Col. Arenal de Guadalupe, Alcaldía Tlalpan, Ciudad de México 14389, Mexico; 4 Unidad de Ingeniería de Tejidos, Terapia Celular y Medicina Regenerativa, Instituto Nacional de Rehabilitación Luis Guillermo Ibarra Ibarra, Calzada México-Xochimilco, No. 289. Col. Arenal de Guadalupe, Alcaldía Tlalpan, Ciudad de México 14389, Mexico; 5 Unidad de Síntesis de Evidencia, Instituto Nacional de Rehabilitación Luis Guillermo Ibarra Ibarra, Calzada México-Xochimilco, No. 289. Col. Arenal de Guadalupe, Alcaldía Tlalpan, Ciudad de México 14389, Mexico; 6 División de Ciencias Básicas, Facultad de Ingeniería, Universidad Nacional Autónoma de México, Circuito exterior S/N, Coyoacán, Ciudad Universitaria, Ciudad de México 04510, Mexico; 7 Departamento de Biotecnología, Universidad Autónoma Metropolitana, Avenida Ferrocarril San Rafael Atlixco, No. 186. Col. Leyes de Reforma 1^a^ Sección, Alcaldía Iztapalapa, Ciudad de México 09310, Mexico; 8 Facultad de Química, Departamento de Alimentos y Biotecnología, Universidad Nacional Autónoma de México, Circuito exterior S/N, Coyoacán, Cd. Universitaria, Ciudad de México 04510, Mexico; 9 Departamento de Reumatología, Hospital General de México Eduardo Liceaga, Dr. Balmis No. 148 Col. Doctores, Alcaldía Cuauhtémoc, Ciudad de México 06720, Mexico; 10 Laboratorio de Materiales, Instituto de Investigación en Materiales, Universidad Nacional Autónoma de México, Circuito exterior S/N, Coyoacán, Cd. Universitaria, Ciudad de México 04510, Mexico

**Keywords:** poly gallic acid, monosodium urate crystals, oxidative stress, inflammatory, inflammasome, computational modeling

## Abstract

**Background.** Gout is an inflammatory disease caused by the deposition of monosodium urate (MSU) crystals, leading to reactive oxygen species (ROS) production and activation of the NLRP3 inflammasome with subsequent IL-1β release. Poly-gallic acid (PGAL), enzymatically synthesized polymer, has demonstrated antioxidant and anti-inflammatory properties; however, its effect on MSU-induced inflammasome activation remains unclear. Thus, this work aims to evaluate the effect of PGAL on oxidative stress and inflammatory responses induced by MSU crystals in THP-1-derived macrophages. **Methods.** Macrophages were pretreated with PGAL and stimulated with MSU crystals. Cell viability, apoptosis, phagocytosis, ROS and NO production, soluble urate levels, and NLRP3 and IL-1β expression were evaluated. Molecular docking and binding free energy calculations were performed to characterize PGAL–MSU interactions. **Results.** PGAL significantly reduced ROS and NO production, and apoptosis. PGAL treatment also decreased MSU crystal phagocytosis and significantly reduced NLRP3 and IL-1β. Computational analysis revealed that PGAL interacts with MSU crystals predominantly through electrostatic interactions, including Na^+^ coordination and hydrogen bonding. **Conclusions.** PGAL attenuates MSU-induced oxidative stress and NLRP3/IL-1β activation, possibly by interfering with crystal internalization. These findings suggest a mechanism based on modulation of crystal–cell interactions and downstream inflammation responses.

## 1. Introduction

Urate is soluble in plasma at concentrations below 6.8 mg/dL (404.6 μmol/L); however, it can precipitate as monohydrated monosodium urate (MSU) crystals when its saturation threshold is exceeded [1,2], playing a key role in the development of gout [3,4]. Gout is a disabling disease, particularly in its chronic stage, whereas the acute stage is characterized by the formation of MSU crystals within joints and soft tissues [5]. MSU crystals exhibit a triclinic structure in which stacked layers of purine rings form needle-like shapes. These crystals play a central role in the inflammatory response following their phagocytosis by macrophages, which occurs through recognition by Toll-like receptors (TLRs). This process subsequently activates intracellular signaling pathways involving the cryopyrin complex or inflammasome (NLRP3). The NLRP3 inflammasome is composed of the NLRP3 protein, the adaptor apoptosis-associated speck-like protein containing a CARD (ASC), and procaspase-1. This cytosolic complex promotes the activation of inflammatory caspases, leading to the cleavage of the precursor of interleukin-1β (pro-IL-1β) into its active form, IL-1β. Upon secretion, IL-1β triggers the acute inflammatory response [6]. MSU crystals act as potent triggers of inflammation by promoting the production of pro-inflammatory cytokines such as IL-6, tumor necrosis factor alpha (TNF-α), and chemokines such as IL-8, which facilitate the recruitment of innate immune cells [7]. Neutrophil activation, like that of macrophages, leads to the production of cytokines, chemokines, reactive oxygen species (ROS), and matrix metalloproteinases [8,9,10], ultimately contributing to cell death. The inflammatory mechanisms induced by MSU crystals also involve activation of nuclear factor kappa B (NF-kB), indicating convergence of multiple signaling pathways that drive the production of IL-1β and TNF-α [11,12], as well as cell death processes such as pyroptosis, necroptosis, and apoptosis [13,14]. Additionally, oxidative stress may be dependent on TLRs and the adaptor protein myeloid differentiation primary response 88 (MyD88), which, together with NF-κB activation, further promotes NLRP3 inflammasome activation [15].

Recognition of MSU crystals involves both TLR-mediated mechanisms and nonspecific interactions with the plasma membrane through electrostatic binding and aggregation within lipid rafts [12,16]. The polyanionic surfaces of MSU crystals facilitate the deposition of complement proteins, promoting opsonization and enhanced phagocytosis [12]. Interaction between MSU crystals and immunoglobulins constitutes an important regulatory mechanism in gout. During the early inflammatory phase, MSU crystals bind to the Fab region of IgG, promoting opsonization, phagocytosis, and amplification of the inflammatory response through lysosomal enzyme release. During the resolution phase, changes in the protein composition of the crystal surface, particularly apolipoprotein B adsorption, suppress phagocytosis and reduce downstream ROS production and pro-inflammatory cytokine secretion [17]. These observations suggest that modifying crystal surface properties may represent a potential strategy for regulating crystal-induced inflammation.

Current gout management includes nonsteroidal anti-inflammatory drugs (NSAIDs), corticosteroids, and colchicine for acute flares, together with urate-lowering therapies, mainly xanthine oxidase inhibitors and uricosuric agents, for long-term disease control. However, these treatments may be associated with adverse effects, contraindications, or limited efficacy in some patients [18], highlighting the need for alternative therapeutic strategies. Several naturally occurring polyphenols, including gallic acid, resveratrol, quercetin, epigallocatechin gallate, and polydatin, have demonstrated antioxidant and anti-inflammatory activities in experimental models of gout [19]. Despite these promising findings, these compounds are low-molecular-weight molecules whose biological activity has been attributed to intracellular signaling modulation. Poly (gallic acid) (PGAL) represents a structurally distinct phenolic polymer, enzymatically synthesized through laccase-mediated polyoxidation of gallic acid using *Trametes versicolor* under environmentally friendly conditions [20,21]. Unlike monomeric gallic acid and other naturally occurring polyphenols, PGAL is a highly water-soluble, stable multiradical polyanion with an approximate molecular weight of 10 kDa, consisting of approximately 60 gallic acid repeating units arranged in a helical architecture [22]. This polymeric architecture provides multiple phenolic and carboxyl functional groups distributed along the polymer backbone, potentially enabling multiple simultaneous physicochemical interactions with the surface of MSU crystals. These structural features suggest that PGAL may not only modulate intracellular oxidative stress but also influence the physicochemical properties of MSU crystal surfaces, thereby interfering with crystal recognition and internalization by macrophages.

Although PGAL has previously demonstrated cytoprotective, antioxidant, and anti-inflammatory effects in different in vitro models [23,24], its direct interaction with MSU crystals has not been investigated at the molecular level. To address this knowledge gap, we combined molecular docking, binding free energy calculations, and experimental analyses to characterize PGAL-MSU interactions and determine whether these interactions influence crystal recognition, macrophage activation, oxidative stress, and inflammasome signaling.

## 2. Materials and Methods

### 2.1. Synthesis and Characterization of MSU Crystals

MSU crystals were synthesized by dissolving uric acid (Sigma-Aldrich Cat. No. U2625, St. Louis, MO, USA) in deionized water at 60 °C. NaOH (0.5 N; Sigma-Aldrich Cat. No. S8045-500G, St. Louis, MO, USA) was added until the solution reached a pH of 8.9 [25,26]. The solution was allowed to stand overnight at room temperature. The crystals were recovered by centrifugation. The precipitate was dried at 60 °C for 48 h. Crystal characterization was performed using polarized light microscopy (Axioscope 40FL Carl Zeiss, Göttingen, Germany) equipped with a first order red compensator. MSU crystals isolated from the synovial fluid of a patient with gout were used as a reference for morphology, size, and birefringence. Scanning electron microscopy (SEM; SM-5900LV microscope JEOL Ltd., Tokyo, Japan). analysis was conducted. Samples were coated with a thin gold layer by cathodic sputtering, and micrographs were obtained using a JSM-5900LV microscope (JEOL Ltd., Tokyo, Japan). X-ray powder diffraction (XRPD) patterns were collected using a Siemens D5000 diffractometer equipped with Diffrac-AT software version 1. (Siemens AG, Berlin, Germany) in Bragg–Brentano geometry, operated with Cu Kα radiation (λ = 1.5418 Å). Data were acquired at room temperature over a 2θ range of 10–70° with a step size of 0.018°. Phase identification was performed by comparing experimental patterns with the International Centre for Diffraction Data (ICDD) database (Card No. 00-031-1890) [27]. Structural integrity was assessed via the Rietveld method using the Profex software suite (v. 5.3.1), which employs the BGMN algorithm (v. 4.2.22) for full-profile fitting [28]. The refinement strategy followed a full-parameter optimization approach; given the low symmetry of the triclinic MSU lattice (Space Group P1^−^), all six unit-cell dimensions (a, b, c, α, β, γ) were refined simultaneously along with the fractional atomic coordinates for all constituent atoms (Na, O, N, C), and the peak profiles were fitted using a Fundamental Parameters approach to account for crystallite size and instrument geometry.

Prior to use in cell culture experiments, MSU crystals were sterilized by dry heat at 180 °C for 2 h (Esterimatic CAISA Mod. 12-27, Mexico City, Mexico). Sterility was verified by bacteriological cultures. Endotoxin contamination was assessed using the *Limulus amebocyte* lysate assay (E-toxate endotoxin standard, Sigma-Aldrich, Cat. E8029-1VL, St. Louis, MO, USA).

### 2.2. Synthesis and Characterization of PGAL

Briefly, 8.5 g of gallic acid was dissolved in 250 mL of acetate buffer solution (250 mM, pH 5) and transferred to a 500 mL three-necked amber round-bottom flask. Twenty milliliters of NaOH (2 M) were added, and the reaction mixture was kept under constant stirring at 25 °C in an oil bath (IKA RCT basic, NC, USA). Once a homogeneous solution was obtained, *Trametes versicolor* laccase was added to achieve a final concentration of 1 U/mL. The reaction was allowed to proceed for 24 h under continuous stirring, with constant oxygenation provided by an air pump (Elite 800, Hagen Inc., QC, Canada). The reaction mixture was added dropwise into 1.25 L of cold ethanol. The precipitate was collected by filtration and vacuum-dried (1.0 × 10^−4^ mbar), yielding a black powder with an approximate yield of 95% [22,29]. The chemical characteristics of PGAL were evaluated using proton nuclear magnetic resonance (^1^H NMR) spectroscopy, size-exclusion chromatography (SEC), dynamic light scattering (DLS), and zeta-potential analysis. ^1^H NMR spectra were acquired on a Varian Unity Inova spectrometer (CA, USA) operating at 400 MHz, using deuterated water as the solvent. The number-average molecular weight (Mn) and molecular- weight dispersity of PGAL were determined by SEC using HPLC equipped with a refractive-index detector (Agilent Technologies, CA, USA). Separation was performed using two Ultrahydrogel 500 columns (7.8 × 300 mm; Waters, MA, USA) connected in series and maintained at 30 °C. The systems were calibrated with polyethylene glycol standard, and ultrapure deionized water containing 0.1 M LiCl was used as the mobile phase at a flow rate of 0.6 mL min^−1^. Before analysis, samples were dissolved in the mobile phase and filtered through 0.45 µm membranes [30]. The zeta particle size polydispersity index (PdI) and DLS were determined using a Zetasizer Nano ZS (Malvern Instruments, Herrenberg, Germany). Measurements were performed in triplicate at 25 °C, with 10 runs per measurement, and PGAL was dispersed in 10 mM NaCl [29].

### 2.3. Cell Culture and In Vitro Model of Acute Inflammation Induced by MSU Crystals

This study was approved by the Research Committee of the Instituto Nacional de Rehabilitación Luis Guillermo Ibarra Ibarra (INRLGII; approval number 110/25). The human monocytic leukemia cell line THP-1 (American Type Culture Collection TIB- 202, Rockville, MD, USA) was used. Cells were maintained in RPMI 1640 medium (Capricorn Scientific, Cat. No, CP24-7598, BW, Germany) supplemented with 10% fetal bovine serum (FBS; Cat. No. 19001, Products, Guadalajara, Jal, Mexico) and 1% penicillin-streptomycin (PS) (Gibco, Cat. No. 15070-063, Grand Island, NY, USA). Cultures were incubated at 37 °C in a humidified atmosphere with 5% CO2 (HERAcell vios 160i LK, Thermo Scientific, HE, Germany). THP-1 differentiation into macrophage-like cells was induced from a stock density of 1 × 10^6^ cells/mL by treatment with phorbol 12-myristate 13-acetate (PMA, Sigma-Aldrich Cat. No. P1585-1MG, St. Louis, MO, USA) at 100 ng/mL for 3 h [31]. Cells were centrifuged, the PMA- containing medium was removed, and cells were allowed to recover overnight in fresh medium.

Differentiated THP-1 cells were used to evaluate phenotypic conversion to macrophages over 24 h under standard culture conditions using reduced-serum medium (2% FBS). These cells were compared with non- differentiated THP-1 controls. Cell morphology was assessed by light microscopy and crystal violet staining (Sigma-Aldrich, Cat. No. C3886-25, St. Louis, MO, USA). Additionally, expression of surface markers CD44 (FITC Mouse Anti-human CD44, BD Pharmingen Biosciences, Cat. 555478, San Jose, CA, USA) and HLA-DR (APC Mouse Anti-human HLA-DR, BD Pharmingen Biosciences, Cat. 559866, San Jose, CA, USA) was assessed by flow cytometry using a BD Accuri C6 cytometer (Becton Dickinson, Franklin Lakes, NJ, USA) and analyzed with FlowJo software (version V10.10.1). IL-1β production and its response to different concentrations of crystals were quantified using a Human IL-1β Standard ABTS ELISA Development Kit (PeproTech, Cat. No. 900-K95, Rocky Hill, NJ, USA).

### 2.4. Experimental Model

PMA-differentiated THP-1 macrophages were used in all experiments and maintained in RPMI 1640 supplemented with reduced-serum medium throughout this study. Control cells were cultured in fresh medium for 48 h. For the MSU group, differentiated macrophages were first incubated in fresh medium for 24 h, after which the medium was replaced with fresh RPMI containing MSU crystals (150 µg/mL) and incubated for an additional 24 h. For PGAL treatment groups, differentiated cells were pretreated with PGAL at 100 µg/mL (P1) or 200 µg/mL (P2) for 24 h. Following pretreatment, the culture medium was completely removed and replaced with fresh medium containing the corresponding PGAL concentration together with MSU crystals (150 µg/mL), and cells were incubated for an additional 24 h. PGAL control groups were treated identically, except that fresh medium contained the same PGAL concentration without MSU crystals during the second 24 h incubation period. The total stimulation time after MSU exposure was 24 h for all groups.

### 2.5. Cell Viability Assay

Cell viability was assessed using crystal violet staining and the MTT assay, with this staining used to estimate the number of adherent cells based on dye retention. Following the experimental treatments, cells were fixed, staining was performed with crystal violet, and absorbance was measured at 590 nm using a microplate reader (iMark^TM^, Bio-Rad, Tokyo, Japan). The amount of retained dye is proportional to the number of adherent cells with intact membranes. Metabolic activity was evaluated using the MTT cell proliferation assay kit (Abcam, Cat. No. ab211091, Cambridge, UK), according to the manufacturer’s instructions. Briefly, after experiments, MTT reagent was added, and cells were incubated for 3 h at 37 °C. Formazan crystals were subsequently solubilized, and absorbance was recorded at 590 nm. Cell viability is expressed as a percentage relative to the untreated control.

### 2.6. Cell Death and Apoptosis

Cell death and apoptosis were analyzed using the Tali^TM^ Apoptosis kit (Annexin V Alexa Fluor^TM^ 488/Propidium Iodide (PI); Invitrogen, Cat. No. A10788, Vienna, Austria) according to the manufacturer’s instructions. After the experimental treatments, cells were harvested, washed with Dulbecco’s phosphate-buffered saline (DPBS 1X; Biowest, Cat. No. X0515, Nuaillé, France), stained with Annexin V and PI, and incubated for 25 min at 37 °C in the dark. Samples were analyzed using flow cytometry (BD FACSAria III, San Jose, CA, USA) and fluorescence microscopy (Floid Cell Imaging Station, Life Technologies Cat. 4471136, Carlsbad, CA, USA).

### 2.7. Phagocytic Activity and Phagolysosomal Acidification Assay

Cell phagocytic activity was assessed using pHrodo Green-*E. coli* BioParticles conjugate (Invitrogen, Thermo Fisher Scientific, Cat. P35366, Eugene, OR, USA), a pH-sensitive fluorescent probe that increases its fluorescence upon particle internalization and phagolysosomal acidification. THP-1-derived macrophages were pretreated with P1 or P2 for 24 h and subsequently incubated with pHrodo™ Green *E. coli* (20 µg/mL) for an additional 24 h at 37 °C. Cells incubated with pHrodo in the absence of PGAL served as the positive control, whereas cells without pHrodo were used to determine autofluorescence. After washing with DPBS, fluorescence was analyzed using a Floid Cell Imaging Station (Life Technologies, USA). Representative images were obtained from five independent experiments. Phagocytic activity was quantified by counting pHrodo-positive fluorescent events and is expressed as the number of fluorescent events per microscopic field.

### 2.8. Internalization of MSU Crystals by Polarized Light Microscopy

PMA-differentiated THP-1 macrophages were exposed to MSU crystals under the experimental conditions described above. Following incubation, cultures were washed with DPBS to remove non-internalized crystals, and cells were gently detached, resuspended, and mounted on glass slides. Intracellular MSU crystals were identified by their characteristic negative birefringence using a polarized light microscope equipped with a first-order red compensator (Axioskop 40 FL, Carl Zeiss). For each experimental condition, at least five random microscopic fields (≥100 cells) were analyzed at 40× magnification. Phagocytosis was expressed as the percentage of cells containing one or more intracellular birefringent crystals [11].

### 2.9. Urate Quantification

Urate concentration was determined using a commercial enzymatic assay (Uric Acid FS Tood’s (TBHBA), DiaSys Diagnostic Systems, Holzheim, Germany). The reaction mixture was prepared according to the manufacturer’s instructions. Culture supernatants were incubated with the reaction reagent for 5 min at 37 °C, and absorbance was measured at 550 nm using a microplate reader (iMark^TM^ Bio-Rad). Urate concentration was calculated from the supplied standard and expressed as mg/dL.

### 2.10. NLRP3 Inflammasome Protein Expression

NLRP3 protein expression was quantified using a Human NLRP3 ELISA kit (Abcam, Cat. No. ab274401 Cambridge, UK) according to the manufacturer’s instructions. Following the experimental treatments, cells were harvested by centrifugation at 500× *g* for 5 min at 4 °C (Eppendorf 5424 R, Hamburg, Germany), washed, and lysed using cell extraction buffer supplemented with protease inhibitors (Complete, Roche, Cat. 11697498001, Mannheim, Germany). Lysis was performed on ice for 20 min. Cell lysates were centrifuged at 18,000× *g* for 20 min at 4 °C, and the supernatant was collected. Total protein concentration was determined using a Micro BCA protein assay kit (Thermo Scientific, Cat. No. 23235, Rockford, IL, USA) and samples were adjusted to the appropriate concentration before analysis. ELISA standards and samples were analyzed in duplicate, and absorbance was measured at 450 nm using a microplate reader (iMark^TM^, Bio-Rad). NLRP3 concentrations were calculated from the standard curve (0–280 ng/mL) and normalized to total protein content. The results are expressed as µg NLRP3 per mg of total protein.

### 2.11. Assessment of Inflammatory Status: Quantification of IL-1β and IL-6

The inflammatory response was assessed by quantifying IL-1β in cell culture supernatants using a Human IL-1β ABTS ELISA Development Kit (PeproTech, Cat. No. 900-K95, USA), and Human IL-6 Standard ABTS ELISA Development Kit (PeproTech, Cat. No. 900-K16+ HRP conjugate, Rocky Hill, NJ, USA). ELISA plates (Wuxi Nest Biotechnology Co., Cat. 515201, Jiangsu, China) were coated overnight with the capture antibody according to the manufacturer’s protocol. After blocking, standards and samples were analyzed in duplicate, followed by incubation with the biotinylated detection antibody and horseradish peroxidase-conjugated streptavidin. The reaction was developed using ABTS substrate, and absorbance was measured at 415 nm using a microplate reader (iMark^TM^, Bio-Rad). IL-1β concentrations were calculated from the standard curve (8–1000 pg/mL), IL-6 (24–1500 pg/mL).

### 2.12. Determination of ROS and Superoxide

Intracellular ROS were evaluated using the fluorescent probe 5- (and-6)-carboxy-2′,7′-dichlorodihydrofluorescein diacetate (carboxy-H2DCFDA), included in the Image-iT™ LIVE Green Reactive Oxygen Species Detection Kit (Invitrogen, Cat. No. I36007, Carlsbad, CA, USA. This probe is oxidized by intracellular oxidants like H_2_O_2_ to form dichlorofluorescein (DCF), which emits green fluorescence (Ex/Em:488/530 nm). Following the experimental treatments, cells were incubated with 25 µM carboxy-H_2_-DCFDA for 20 min at 37 °C in the dark. After washing, fluorescence images were acquired using a Floid Cell Imaging Station, and quantitative analysis was performed using a Tali^®^ Image-Based Cytometer (Invitrogen, Cat. No. T10791, Carlsbad, CA, USA). Superoxide (O_2_^•^) production was assessed using dihydroethidium (DHE, Invitrogen, Thermo Fisher Scientific Cat. No. D11347, Waltham, MA, USA), which is oxidized by intracellular O_2_^•^ to form ethidium that emits red fluorescence (Ex/Em: 518/605 nm). Cells were incubated with 1.5 µM DHE for 20 min at 37 °C in the dark, and fluorescence was acquired under the same imaging conditions and quantified using the Tali^®^ Image-Based Cytometer. Results were normalized to the control and expressed as relative fluorescence units (RFU).

### 2.13. RNS Analysis

NO production was evaluated using the fluorescent probe 4-amino-5-methylamino-2′,7′-difluorofluorescein diacetate (DAF-FM diacetate; Invitrogen, Thermo Fisher Scientific, Cat. D23842, Carlsbad, CA, USA). This probe reacts with NO-derived species to form a fluorescent benzotriazole derivative (Ex/Em: 495/515 nm). Cells were loaded with 5 µM DAF-FM for 30 min at 37 °C in the dark. Fluorescence was documented using a Floid Cell Imaging Station, acquiring at least three random fields per condition under the same imaging settings. For image-based quantification, fluorescent events were analyzed using automated image analysis and expressed as the number of fluorescent events per field. Additionally, fluorescent cells were quantified using a Tali^®^ Image-Based Cytometer, and results were expressed as RFU.

### 2.14. Statistical Analysis

Differences between non-activated and PMA-stimulated THP-1 cells were assessed using a two-tailed unpaired Student’s *t*-test. Data are presented as mean ± standard deviation (SD), and statistical significance was considered at *p* < 0.05. Quantitative data, including cell viability, apoptosis, necrosis, phagocytosis, ROS, RNS, inflammatory markers, and protein expression, were analyzed using GraphPad Prism V.10 (GraphPad Software, San Diego, CA, USA). A one-way ANOVA was performed, as well as Tukey’s post hoc test, to determine significant differences between means, with *p* ≤ 0.05 considered significant.

### 2.15. Zeta-Potential Analysis of PGAL, MSU Crystals, and PGAL–MSU

The electrokinetic properties of PGAL, MSU crystals, and PGAL–MSU mixtures were evaluated using zeta-potential analysis under two independent experimental conditions, using distilled water as the dispersant. In the first experimental series, non-incubated dispersions consisted of distilled water (control), MSU crystals at 500 µg/mL, P1 or P2, and mixtures of PGAL at either concentration with crystals. In a separate experimental series, dispersions consisted of control, MSU crystals at 150 µg/mL, P1 or P2, and mixtures of PGAL at either concentration with MSU crystals at 150 µg/mL. These samples were incubated for 72 h at 37 °C under controlled cell-culture conditions before analysis. Zeta potential was measured using a Zetasizer Nano ZS (Malvern Instruments Ltd., Malvern, Worcestershire, UK) equipped with a zeta dip cell, at a measurement temperature of 25 °C. Each condition was analyzed in technical triplicate, and the results are expressed as mean ± SD.

### 2.16. Crystallography-Derived Structural Modeling

**Figure 1:**
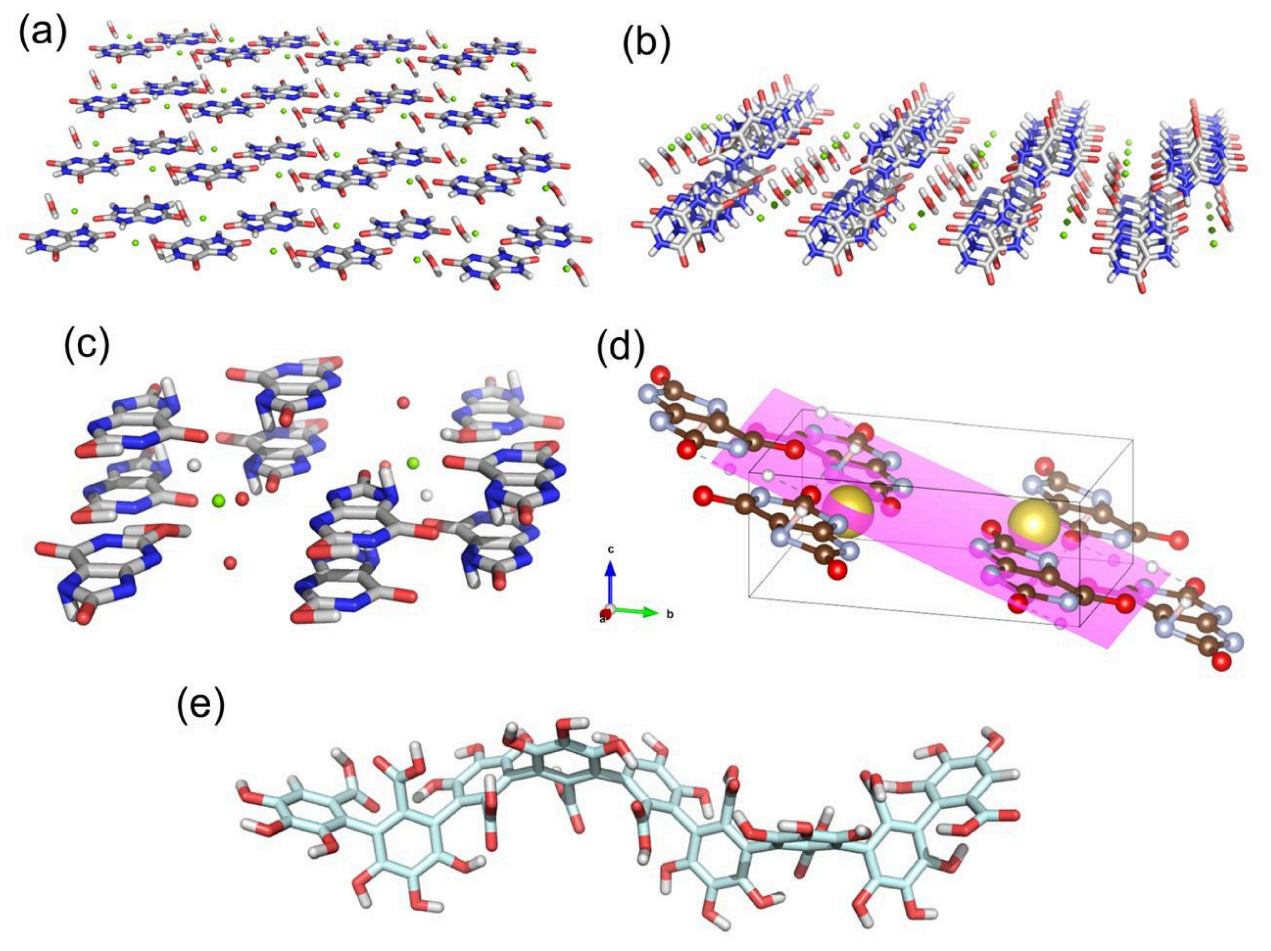
Structural models of MSU crystals and PGAL polymer. (**a**–**c**) Representative views of the extended crystalline lattice of MSU monohydrate showing the molecular stacking and the distribution of sodium ions (green/small spheres) and water molecules within the network. (**d**) Detailed atomic structure of the MSU unit cell (black wireframe). The (001) crystallographic plane is highlighted in translucent purple, demonstrating the spatial arrangement of the urate molecules and sodium ions (yellow spheres) relative to this specific plane within the triclinic lattice P1^−^. (**e**) PGAL chain, illustrating the periodic arrangement of aromatic rings and functional groups hydroxyl and carboxyl along the polymeric backbone.

The crystallographic parameters obtained using Rietveld refinement of the XRPD data were used to generate a three-dimensional model of MSU based on its triclinic crystal structure. Atomic coordinates were retrieved from the Cambridge Crystallographic Data Centre (CCDC, deposition No. 1217343) [27,32], and structural visualization and model construction were performed using VESTA software (version 3.5.8; Koichi Momma and Fujio Izumi, Tsukuba, Japan)**.** (Figure 1a). Slab models corresponding to the (110) and (011) crystallographic planes were generated based on previously reported structural analysis [33,34] and exported as PDB files for subsequent molecular docking studies (Figure 1b). The (001) plane did not show favorable electrostatic interaction with PGAL under this screening and was therefore excluded from subsequent Poisson-Boltzmann free-energy calculations, where the (010) and (011) planes showed favorable interactions and were selected for detailed energetic analysis. In addition, the biological relevance of the (011) plane is supported by previous crystallographic studies identifying this facet as an exposed plane of needle shaped MSU crystals isolated from synovial fluid [33]. The computational model of PGAL used for molecular docking consisted of nine-unit gallic acid oligomer with explicit hydrogen atoms, based on a previously optimized structure. The geometry was optimized at the M06-2X/6-311++G (d,p) level of theory (Figure 1c) [22].

### 2.17. Molecular Docking of MSU-PGAL Complexes

Molecular docking simulations were performed to investigate the interaction between PGAL and the MSU crystal surface. Docking calculations were carried out using the entire MSU crystal model to explore potential binding orientations. A total of 100 poses were generated, and those exhibiting electrostatic interactions with Na^+^ ions were selected for further analysis. The selected complexes were subsequently processed using the PDB2PQR server to assign atomic charges and atomic radii, generating PQR files suitable for electrostatic calculations, whereas the Gasteiger partial charges used during docking preparation were retained. These structures were then used to evaluate the electrostatic and non-electrostatic contributions to the binding free energy between PGAL and the MSU crystal surfaces.

### 2.18. Binding Free Energy Calculations

The binding free energy (*ΔG_b_*) of the MSU-PGAL complexes was calculated using the methodology previously described [35]. The total binding free energy was decomposed into electrostatic (*ΔG_elec_*) and non-electrostatic (*ΔG_non-elec_*) contributions according to the following equations:

(1) $$\Delta G_{b}\;=\;\Delta G_{elec}\;+\;\Delta G_{non-elec}$$

and

(2) $$\Delta G_{elec}=\Delta G_{coul}+\Delta G_{solv}$$

*ΔG_b_* is expressed as a function of both electrostatic and non-electrostatic energy (*ΔG_elec_* and *ΔG_non-elec_*, respectively). Accordingly, the total binding free energy can be expressed as

(3) $$\Delta G_{b}=\Delta G_{coul}+\Delta G_{dolv}+\Delta G_{non-elec}$$

*ΔG_elec_* depends on both Coulombic energy (*ΔG_coul_*) and solvation energy (*ΔG_solv_*). Electrostatic contributions were calculated using the Adaptive Poisson-Boltzmann Solver (APBS) [35], which numerically solves the Poisson-Boltzmann equation. Atomic charges were assigned using AutoDock Tools 4 [36,37]. Calculations were performed using a dielectric constant of 78 for water [38] and 3.0 for PGAL. The dielectric constant of PGAL has not been explicitly reported; it was estimated based on structural similarities to other organic polymers, whose dielectric constants typically range between 2.0 and 4.0. Therefore, a value of 3.0 was selected as a reasonable approximation, consistent with reported values for related polymeric systems [39]. Electrostatic contributions were calculated under physiological pH conditions using an ionic strength of 0.15 M to account for electrostatic screening effects. Mobile ions were treated implicitly through the Poisson-Boltzmann continuum model.

The non-electrostatic contribution (*ΔG_non-elec_*) to *ΔG_b_* was estimated from the solvent-accessible surface area (ASA) according to Equation (4), using a surface tension coefficient (γ) of 0.021 kJ mol^−1^ Å^−2^

(4) $$\Delta G_{non-elec}=\gamma \;\left(\Delta {ASA}_{PGAL-MSU}\right)-\Delta {ASA}_{PGAL}-\Delta {ASA}_{MSU}$$

ASA calculations were performed using Visual Molecular Dynamics (VMD) [40] for both the PGAL-MSU complexes and the isolated molecules. The resulting electrostatic and non-electrostatic terms were combined to obtain the total binding free energy (*ΔG_b_*).

## 3. Results

### 3.1. Characterization of MSU Crystals

**Figure 2:**
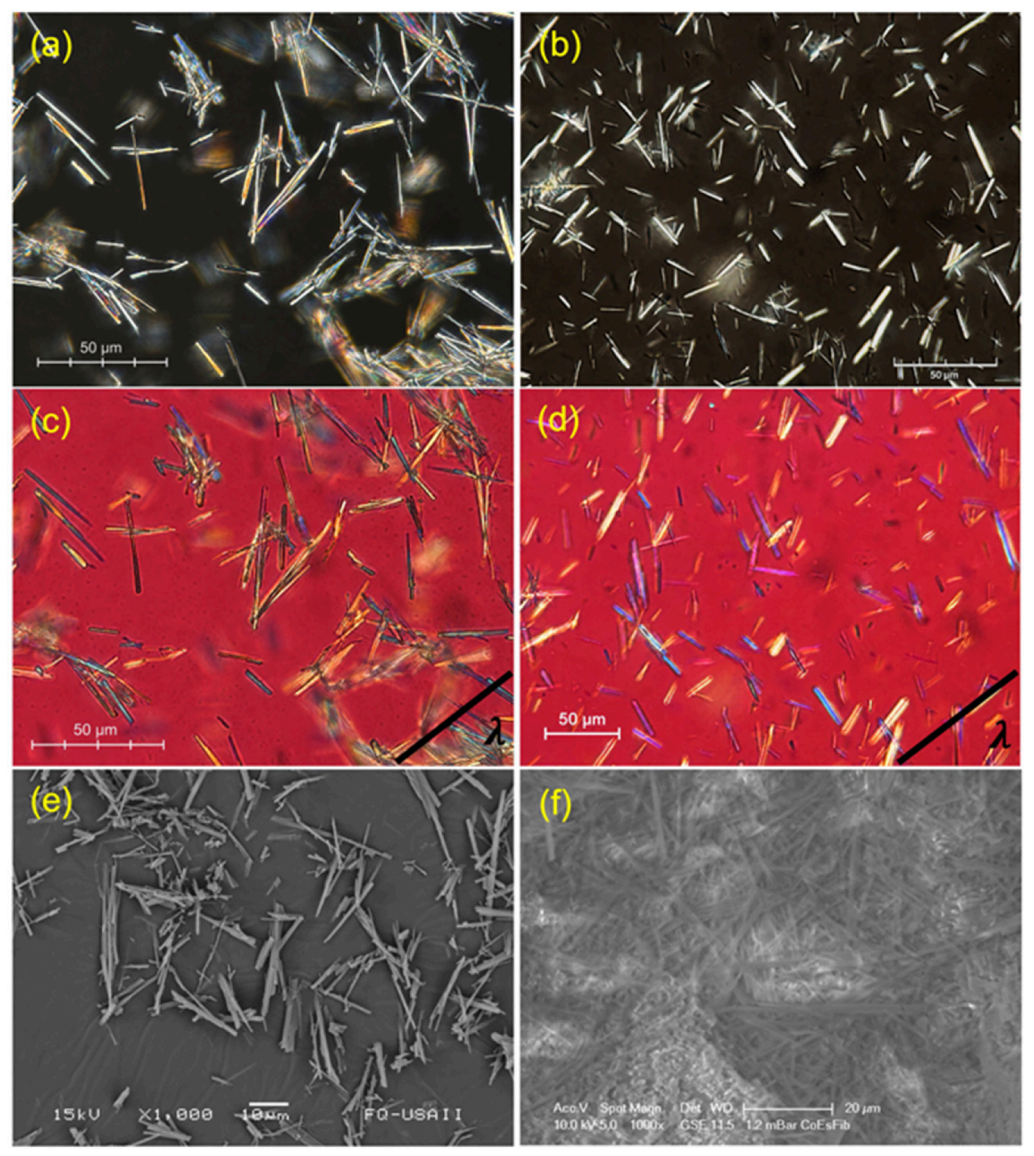
Morphological and structural characterization of synthetic and clinical MSU crystals. (**a**) Polarized light micrograph (40× objective) of synthetic MSU crystals. (**b**) Polarized light micrograph of MSU crystals isolated from the synovial fluid of a patient with gout. (**c**) Synthetic MSU crystals observed under compensated polarized light with a first-order red (λ) compensator. (**d**) MSU crystals from synovial fluid observed under compensated polarized light. (**e**) SEM image of synthetic MSU crystals, illustrating well-defined acicular morphology. (**f**) SEM image of MSU crystals isolated from synovial fluid, showing increased crystal aggregation and amorphous material.

Polarized light microscopy demonstrated that synthetic MSU crystals exhibited the characteristic needle-shaped morphology and negative birefringence of MSU, closely resembling crystals isolated from the synovial fluid of patients with gout. SEM analysis showed that synthetic crystals displayed a uniform acicular morphology with smooth surfaces, whereas clinically derived crystals exhibited greater heterogeneity, aggregation, and amorphous material, likely associated with synovial fluid components (Figure 2).

The XRPD analysis confirmed the presence of highly crystalline monosodium urate monohydrate (MSU) in both synthetic and patient-derived samples, showing patterns consistent with reference data [27,41]. Supplementary Figure S1a displays the diffraction patterns of the MSU samples, where all reflections perfectly align with the expected triclinic phase. The structural parameters were further validated through a comprehensive Rietveld refinement (Supplementary Figure S1b), which reached stable convergence after 12 iterations. The refinement yielded a weighted-profile R-factor (Rwp) of 11.85% and a goodness-of-fit (χ^2^) of 5.52, indicating a robust agreement between the observed and calculated patterns (Table 1).

The refined lattice parameters (a = 10.8805(4) Å, b = 9.5148(5)Å, c = 3.5650(2)Å) and angles show minimal deviation from literature values, confirming the structural fidelity of the synthesized phase. The analysis also revealed a pronounced intensity in the (011) reflection, significantly higher than other planes in all samples. This observation suggests a marked preferential orientation along the [011] crystallographic direction, consistent with the characteristic needle-like growth habit of MSU crystals. The mean crystallite size associated with this reflection was estimated at 63.7 ± 0.6 nm using the Scherrer equation.

### 3.2. Characterization of PGAL

The ^1^H NMR spectrum of PGAL showed only the residual HDO signal, with no resolved resonances attributable to free gallic acid under the analytical conditions used (Supplementary Figure S2). SEC analysis yielded a Mn of 5644 ± 95 Da and a molecular-weight dispersity (Đ) of 1.946. DLS analysis showed a PdI of 0.751 ± 0.521, while zeta-potential analysis in 10 mM NaCl yielded −1.561 ± 1.71 mV.

### 3.3. PMA-Induced Differentiation of THP-1 Cells into Macrophages

Untreated THP-1 cells exhibited a rounded morphology, grew in suspension, and formed cell aggregates, consistent with a monocyte phenotype. Following stimulation with PMA, cells underwent a morphological transition to an adherent phenotype, characterized by increased cell size, irregular contours, and the presence of cytoplasmic extensions. CD44 expression was increased slightly in PMA-treated cells (82.5 ± 10.9%) compared to non-treated cells (74.8 ± 20.5%), suggesting greater adhesion and activation capacity associated with the macrophage phenotype; however, this increase was not significant. HLA-DR expression was significantly reduced in PMA-treated cells (9.20 ± 6.25%) compared to non-treated cells (31.61 ± 9.38%) (** *p* < 0.01). PMA stimulation significantly increased basal IL-1β levels compared with undifferentiated THP-1 cells (** *p* < 0.01). In the acute inflammation model, exposure to MSU crystals further increased IL-1β expression relative to differentiated cells not exposed to crystals (* *p* < 0.05). However, a concentration-dependent trend was observed, with higher crystal concentrations inducing greater IL-1β production (Supplementary Figure S3).

### 3.4. Effect of PGAL on Cell Viability

**Figure 3:**
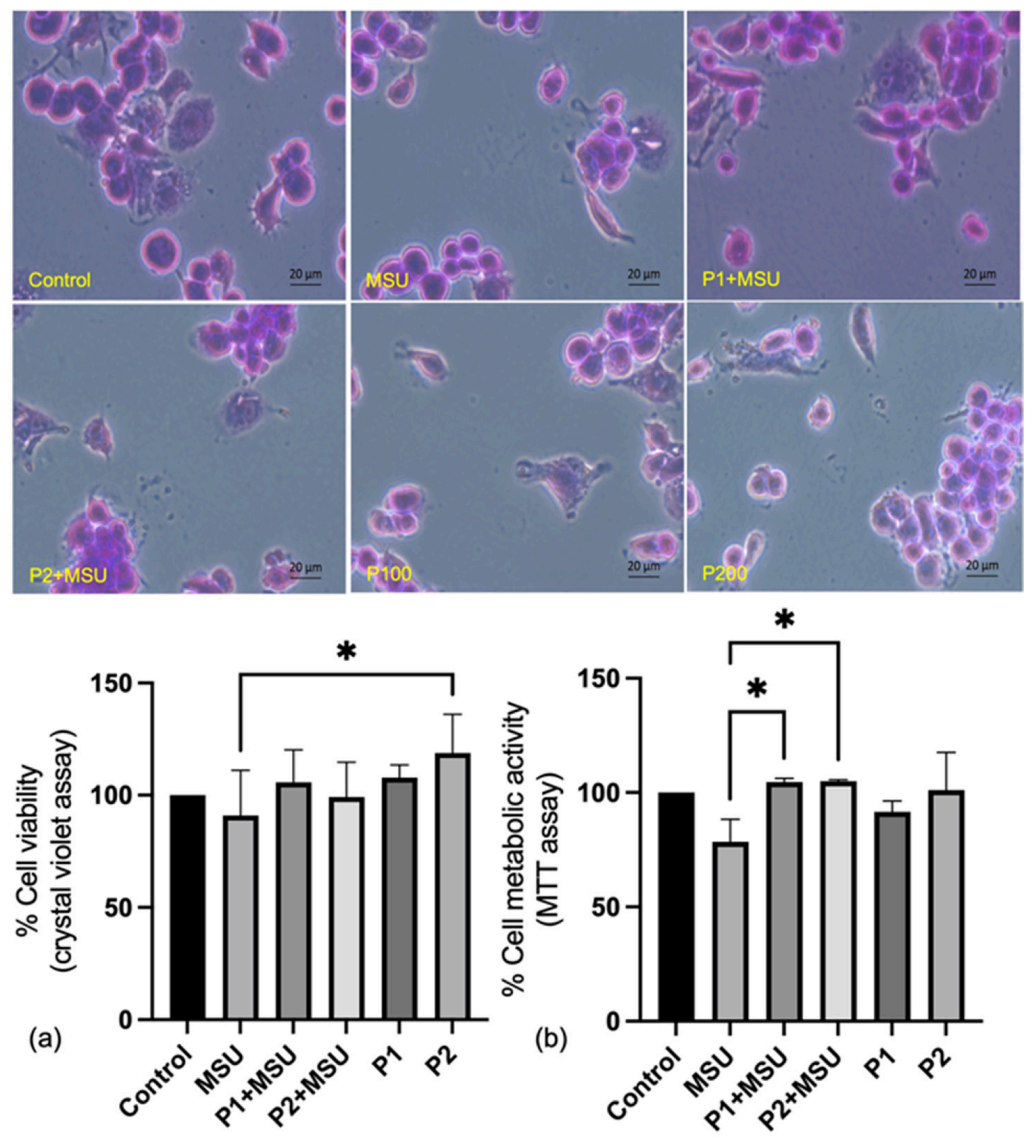
Effect of PGAL on cell viability and metabolic activity in THP-1 cells treated with MSU crystals. Representative micrographs of crystal violet-stained cells after 24 h of treatment. (Control) unstimulated cells; (MSU) cells stimulated with MSU crystals; (P1 + MSU and P2 + MSU) cells treated with PGAL at 100 and 200 µg/mL, respectively, with MSU; (P100 and P200) cells treated with PGAL at 100 and 200 µg/mL. Scale bar: 20 µm. Lower panels show quantification of cell viability: (**a**) crystal violet staining (absorbance normalized to control); and (**b**) metabolic activity determined by the MTT assay (% of control). Data are expressed as mean ± SD from five independent experiments (n = 5). Data are shown as individual values with mean ± SD. * *p* < 0.05.

Control THP-1 cells exhibited high cell density, preserved morphology, and strong adherence to the culture surface. In contrast, exposure to MSU crystals resulted in reduced cell density, accompanied by cytoplasmic retraction and partial loss of adhesion. Treatments with PGAL improved the preservation of cell morphology compared to MSU-treated cells, with no evident morphological alterations indicative of cytotoxicity in PGAL-treated groups. Crystal violet staining showed no statistically significant differences in adherent cell density among the control (100%), MSU crystals (90.88 ± 20.17%), P1 + MSU (105.71 ± 14.51%), and P2 + MSU (99.16 ± 15.50%) groups. PGAL treatment alone did not significantly affect cell density, indicating no detectable cytotoxic effect under the evaluated conditions. By contrast, the MTT assay showed that exposure to MSU crystals reduced metabolic viability to 78.47 ± 9.88% of the control value. Treatment with PGAL significantly increased the metabolic activity compared with the MSU group, reaching 104.60 ± 1.53 with P1 + MSU crystals (*p* = 0.0272) and 104.88 ± 0.66 with P2 + MSU crystals (*p* = 0.0488). PGAL treatment alone did not significantly alter the metabolic activity relative to the control (Figure 3).

### 3.5. Effect of PGAL on Apoptosis

Exposure to MSU crystals was associated with a numerical reduction in the proportion of viable cells (No stained: Annexin V^−^/EthD-1^−^) to 54.61 ± 25.23%, compared to 76.68 ± 7.45% in the control group. However, this difference did not reach statistical significance. Similarly, the Annexin V^+^/EthD-1^−^ population numerically increased from 6.61 ± 9.93% in control cells to 23.08 ± 6.98% following MSU treatment. In contrast, the Annexin V^+^/EthD-1^+^) population numerically increased from 2.53 ± 0.74% in the control to 26.86 ± 14.67% following MSU exposure (*p* < 0.05). The proportion of EthD-1^+^ dead cells (Annexin V−/EthD-1^+^) decreased from 22.44 ± 9.45% in the control to 3.52 ± 2.10% after the MSU exposure (*p* < 0.01). PGAL treatment resulted in numerical increases in the viable cell population to 59.03 ± 15.29% and 68.09 ± 16.85% at concentrations of 100 µg/mL (P1 + MSU) and 200 µg/mL (P2 + MSU), respectively, compared with the MSU-treated; however, these differences were not statistically significant. Similarly, the Annexin V^+^/EthD-1^−^ population numerically decreased to 19.30 ± 11.39 and 14.22 ± 8.28% following treatment with P1 + MSU, P2 + MSU, respectively, without reaching statistical significance. Both PGAL concentrations significantly reduced the Annexin V^+^/EthD-1^+^ population to 3.35 ± 2.60% and 3.89 ± 3.39%, respectively, compared with the MSU-treated group (*p* < 0.05). (Supplementary Figure S4).

### 3.6. Effect of PGAL on Acidification-Dependent Phagocytosis Activity and MSU Crystal Internalization

**Figure 4:**
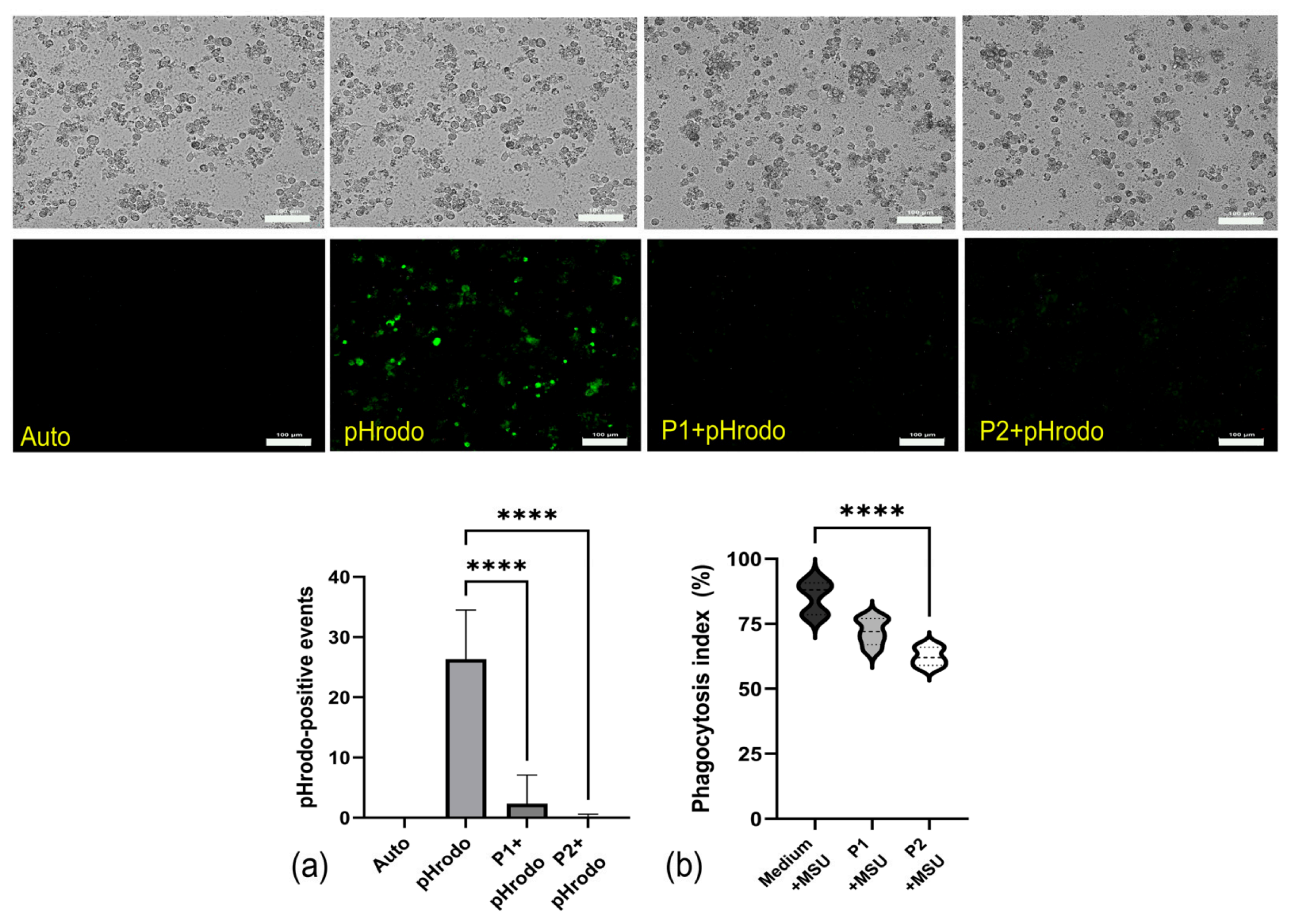
Effect of PGAL on acidification-dependent phagocytosis and MSU crystal internalization. Upper panel: bright-field images showing cell density and morphology under different experimental conditions. Representative images from one of six independent experiments are shown. Middle panel: representative fluorescence micrographs following incubation with pHrodo™ Green *E. coli* BioParticles. Green fluorescence indicates intracellular internalization and acidification. (**a**) Quantification of fluorescent events per field obtained by automated image analysis (n = 6). (**b**) Quantification of MSU crystal phagocytosis by polarized light microscopy. Data are expressed as mean ± SD (**** *p* < 0.0001).

Cells incubated with pHrodo conjugated *E. coli* bioparticles exhibited a strong punctate fluorescent signal, consistent with active particle internalization and acidification of endosomal and phagolysosomal compartments. PGAL treatment significantly reduced the pHrodo-positive signal, with decreases of 91.14 ± 4.76% and 99.37 ± 0.40% in pHrodo-positive events for P1 and P2, respectively, compared with cells exposed to pHrodo alone (**** *p* < 0.0001) (Figure 4a). Consistent with these findings, polarized light microscopy showed reduced MSU crystal internalization following PGAL treatment. Compared with the MSU group (85.3 ± 6.37% of cells containing intracellular crystals), P1 reduced crystal internalization by 13.3 ± 5.19%, although this difference was not statistically significant, whereas P2 produced a significant reduction of 22.9 ± 3.64% (**** *p* < 0.0001) (Figure 4b).

### 3.7. Effect of PGAL on Soluble Urate Levels, NLRP3 Protein Expression, and Secretion of Pro-Inflammatory Cytokines

Exposure to MSU crystals significantly increased soluble urate levels compared to the control group (6.41 ± 0.99 vs. 0.46 ± 0.19 mg/dL; *p* < 0.0001). Soluble urate concentrations were numerically lower in the P1 + MSU (5.31 ± 0.20 mg/dL) and P2 + MSU (4.86 ± 1.45 mg/dL) groups than in the MSU group; however, neither difference was statistically significant. PGAL treatment alone did not significantly alter baseline soluble urate concentrations (Supplementary Figure S5).

**Figure 5:**
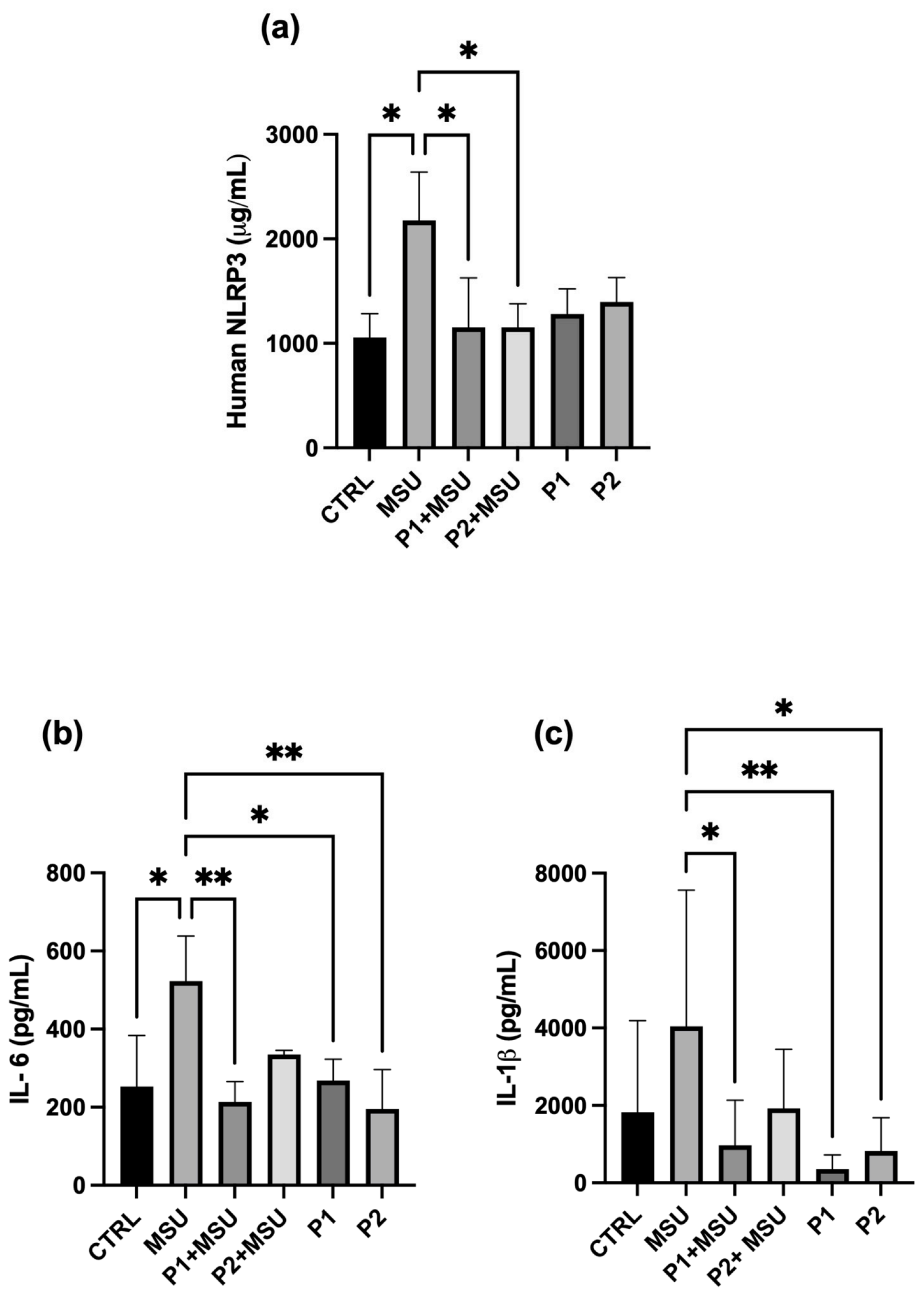
Effect of PGAL on inflammasome expression and pro-inflammatory cytokine production. (**a**) MSU crystals increased NLRP3 levels compared to control (* *p* < 0.05). PGAL treatment reduced MSU-induced inflammasome expression. (**b**) IL-6 production. (**c**) IL-1β production. Data are expressed as mean ± SD. Statistical analysis was performed using one-way ANOVA followed by Tukey’s post hoc test * *p* < 0.05, ** *p* < 0.01.

MSU stimulation significantly increased NLRP3 protein expression compared with the control (2177.0 ± 378 vs. 1055.3 ± 228 µg/mg total protein; *p* = 0.0127). Both PGAL treatments significantly reduced MSU-induced NLRP3 expression by approximately 47%, with values of 1153.7 ± 385 µg/mg for P1 + MSU (*p* = 0.0236) and 1154.2 ± 224 µg/mg for P2 + MSU (*p* = 0.0237), compared with the MSU group. PGAL treatment alone did not significantly alter NLRP3 expression relative to the control (Figure 5a). MSU crystal exposure significantly increased IL-6 levels (523.25 ± 114.97 *p* = 0.0252) compared with the control group (253.17 ± 130.76). Treatment with P1 significantly reduced MSU-induced IL-6 levels to 213.88 ± 51.63 (*p* = 0.0100), whereas the numerically lower value observed with P2 + MSU (334.92 ± 10.76) did not reach statistical significance (*p* = 0.1639 vs. MSU). PGAL treatment alone did not significantly alter IL-6 levels compared with the control (Figure 5b). IL-1β concentrations were numerically higher in MSU-stimulated cells (4041.22 ± 3521.61 pg/mL) compared to the control (1821.38 ± 2368.19 pg/mL), although this difference was not statistically significant. P1 + MSU significantly reduced IL-1β levels (971.48 ± 1167.57 pg/mL; *p* = 0.0182) compared with the MSU crystals group, whereas the lower value observed with P2 + MSU (1921.55 ± 1527.37 pg/mL) did not reach statistical significance (*p* = 0.2070). PGAL treatment alone significantly reduced IL-1β concentrations compared with the MSU group (Figure 5c).

### 3.8. PGAL Modulates MSU Crystals-Induced ROS Production

**Figure 6:**
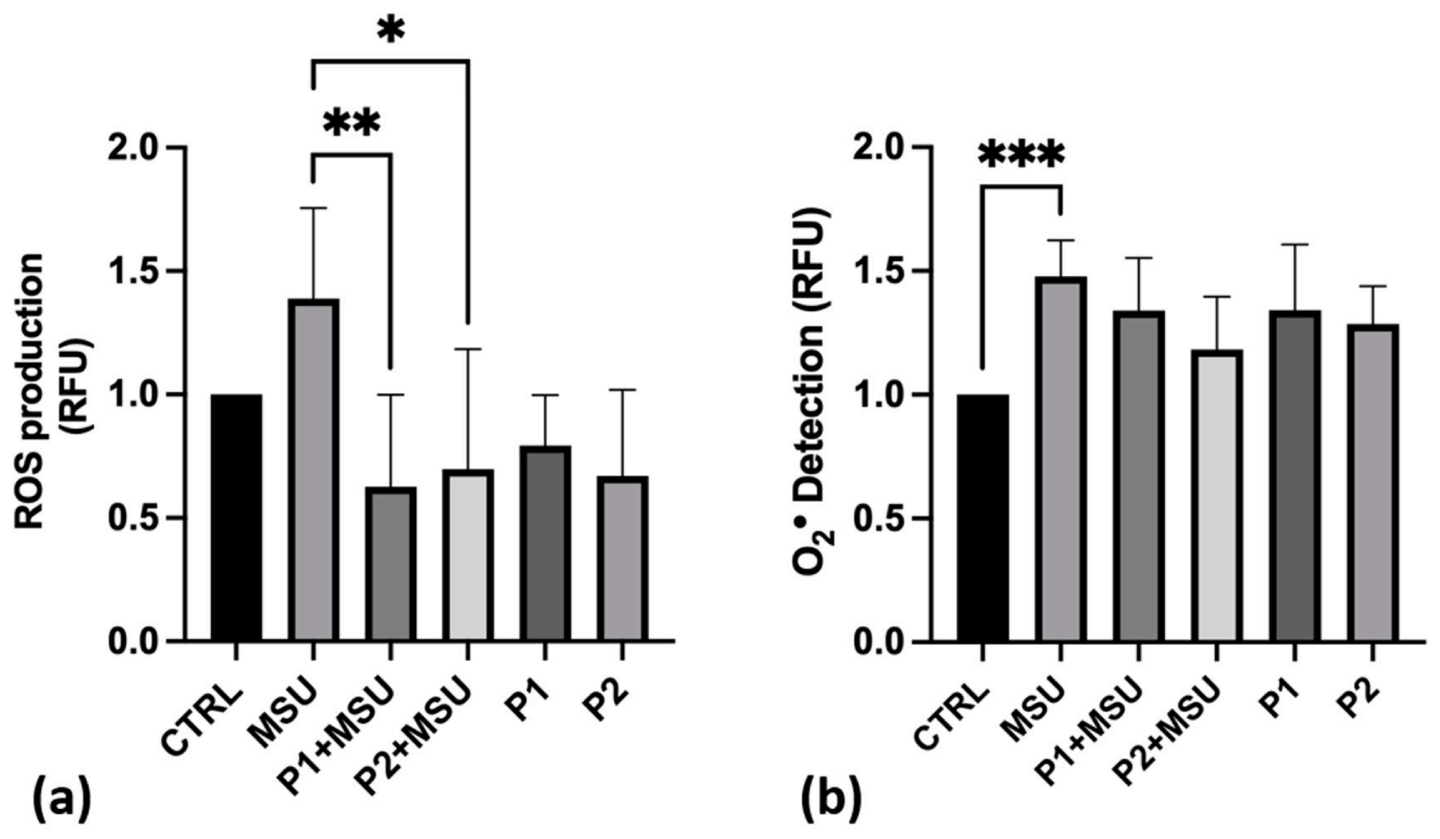
Effect of PGAL on MSU-induced ROS production. (**a**) Detection of intracellular ROS using DCFH-DA oxidation (green fluorescence, Ex/Em: 488/530 nm). (**b**) Detection of superoxide (O_2_^•−^) using dihydroethidium (DHE) oxidation to ethidium (red fluorescence, Ex/Em: 518/605 nm). Quantification was performed using image-based cytometry. Data are expressed as mean ± SD. Statistical analysis was performed using one-way ANOVA followed by Tukey’s post hoc test * *p* < 0.05; ** *p* < 0.01, *** *p* < 0.001.

**Figure 7:**
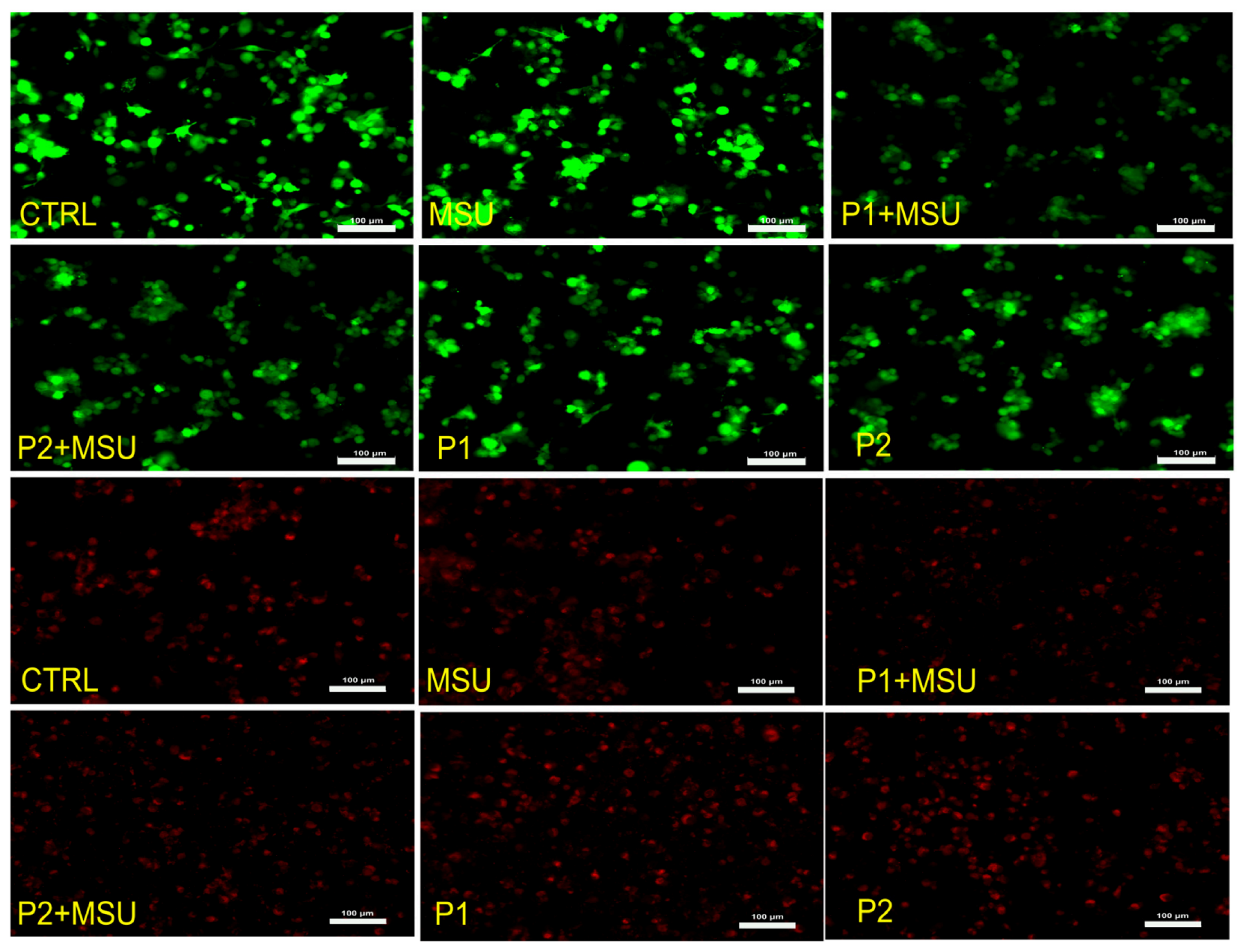
Representative images show increased fluorescence following MSU stimulation, while PGAL treatment reduces the signal in both assays. (**Upper panel**) Detection of intracellular ROS using carboxy-H2DCFDA (green fluorescence, Ex/Em: 488/530 nm). (**Lower panel**) Detection of superoxide (O_2_^•−^) using dihydroethidium (DHE) (red fluorescence, Ex/Em: 518/605 nm).

Exposure to MSU crystals increased intracellular ROS levels (1.38 ± 0.36 RFU) compared with the control group, as indicated by increased fluorescence intensity. PGAL treatment significantly reduced ROS levels in MSU-stimulated cells. Specifically, ROS levels decreased to 0.62 ± 0.37 RFU in the P1 + MSU group (*p* = 0.0055) and 0.69 ± 0.48 RFU in the P2 + MSU group (*p* = 0.0142). PGAL treatment alone did not significantly alter basal ROS signal (P1: 0.79 ± 0.20 RFU; P2: 0.66 ± 0.34 RFU) relative to the control (Figure 6a and Figure 7).

O_2_^•^ levels were significantly higher in MSU-stimulated cells (1.46 ± 1.00 RFU) than in control cells (1.00 RFU; *p* = 0.0004). Numerically lower O_2_^•^ levels were observed in the P1 + MSU (1.19 ± 0.45 RFU) and P2 + MSU (1.09 ± 0.35 RFU) groups; however, neither treatment differed significantly from the MSU crystals group (*p* > 0.05). PGAL treatments alone did not significantly alter basal O_2_^•^ levels (Figure 6b and Figure 7).

### 3.9. PGAL Reduces MSU-Associated RNS Production

**Figure 8:**
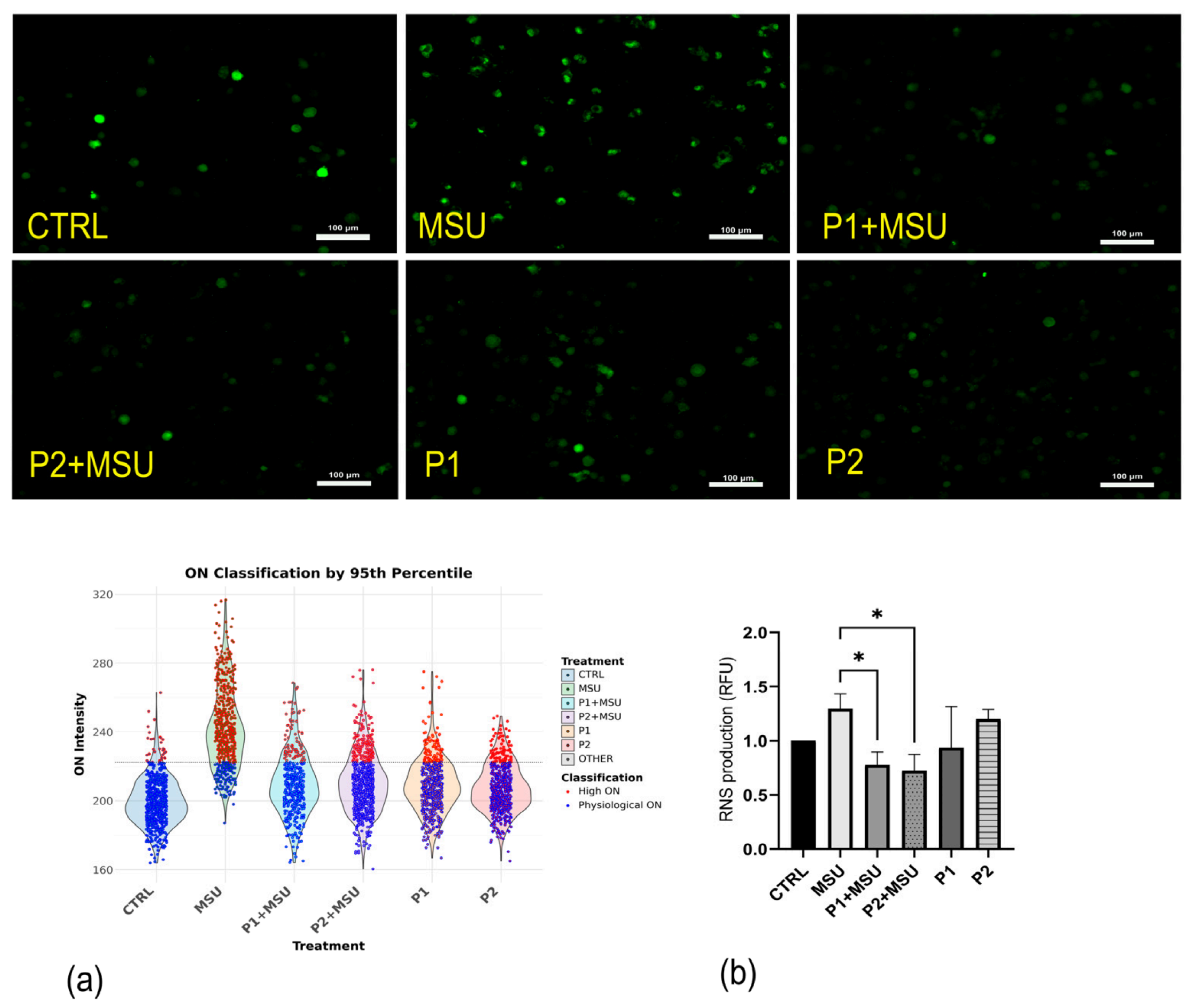
Modulation of NO production in MSU-stimulated THP-1 cells by PGAL. Representative fluorescence micrographs showing intracellular NO production (green fluorescence). Scale bar: 100 µm. (**a**) Global quantification of fluorescence intensity, including classification of high-intensity events using the 95th percentile threshold to identify NO-producing cells. (**b**) Image-based cytometry analysis showing the distribution of fluorescence intensity normalized to the control group. Data are expressed as mean ± SD from independent experiments * *p* < 0.05.

PGAL treatment significantly reduced NO-associated fluorescence in MSU-stimulated cells. Compared with the MSU group (1.29 ± 0.13 RFU), fluorescence decreased to 0.77 ± 0.11 RFU in the P1+ MSU crystals group (*p* = 0.0338) and to 0.72 ± 0.14 RFU in the P2 + MSU cells (*p* = 0.0280). No significant difference was observed between the two PGAL concentrations. PGAL treatment alone did not significantly alter basal NO-associated fluorescence relative to the control. The MSU group showed a numerically higher fluorescence signal than the control; however, this difference was not statistically significant (Figure 8).

### 3.10. Electrokinetic Behavior of MSU Crystals, PGAL and PGAL–MSU Mixed

Zeta-potential analysis was used to characterize the electrokinetic behavior of MSU crystals, PGAL and PGAL–MSU dispersions under two independent experimental conditions. In the non-incubated series, MSU crystals exhibited a zeta potential of −31.27 ± 1.04 mV, whereas P1 showed values of −24.53 ± 4.81 and P2 values of −31.37 ± 1.45 mV. When PGAL was combined with crystals, the resulting dispersions exhibited zeta potentials of −32.23 ± 1.99 (P1 + MSU) and −32.17 ± 1.39 mV at P2 with MSU. Thus, both mixed dispersions retained negative zeta-potential values comparable to those of MSU crystals alone. In a separate experimental series incubated for 72 h under controlled conditions, MSU crystals exhibited a zeta potential of −20.57 ± 6.55 mV. PGAL alone showed more negative values of −34.43 ± 5.00 and −40.43 ± 9.95 mV at P1 and P2, respectively. By contrast, PGAL–MSU mixed dispersions exhibited values of −21.23 ± 6.03 (P1 + MSU) and −20.60 ± 3.92 mV (P2 + MSU), remaining close to that of MSU crystals alone under the same experimental conditions (Table 2).

Table 2 Data are presented as mean ± SD of three technical measurements. MSU, monosodium urate crystals; P1, PGAL at 100 µg/mL; P2, PGAL at 200 µg/mL; P1 + MSU and P2 + MSU, the respective PGAL concentrations combined with MSU crystals. MSU was used at 500 µg/mL in the non-incubated aqueous series and at 150 µg/mL in the series incubated for 72 h at 37 °C, 95% relative humidity, and 5% CO_2_.

### 3.11. In Silico Interaction Between MSU Crystals and PGAL: Molecular Docking and Binding Free Energy Calculations

**Figure 9:**
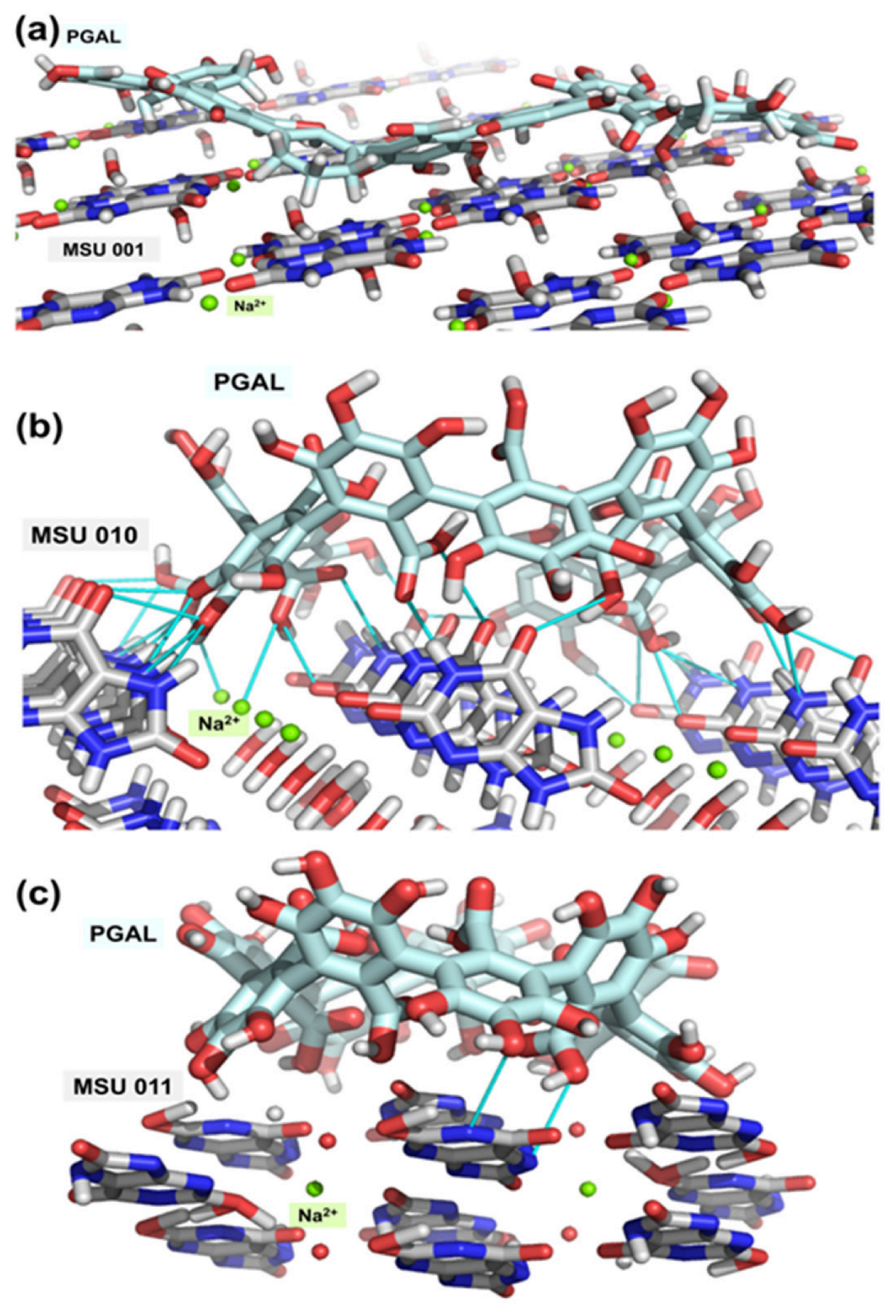
Molecular interaction between PGAL and different MSU crystal planes. (**a**) PGAL on the MSU (001) plane, showing electrostatic interactions with Na^+^ ions and few hydrogen bonds (cyan lines). (**b**) PGAL on the MSU (010) plane, exhibiting the highest number of hydrogen bonds. (**c**) PGAL on the MSU (011) plane, displaying fewer hydrogen bonds and electrostatic interactions with Na^+^ ions.

Molecular docking simulations were performed to evaluate the interaction between PGAL and different MSU crystal planes (001, 010, and 011). The resulting binding poses are shown in Figure 9, where distinct interaction patterns can be observed depending on the crystallographic orientation of the urate plane. The calculated *ΔG_b_* values should therefore be interpreted as the electrostatic contribution to PGAL adsorption rather than absolute binding free energies, since the Poisson-Boltzmann approach employs a continuum electrostatic model that is most appropriate for comparative analyses of crystallographic surface affinity [35]. Future molecular dynamics simulations incorporating explicit solvent, physiological ionic strength, counterions, and appropriate protonation states could provide a more comprehensive evaluation of the stability of the predicted PGAL-MSU interactions under physiological conditions.

To quantitatively assess the stability of the complexes, binding free energy calculations were performed [35,40], and the results were summarized in Table 3. The calculated energy components included solvation energy (*ΔG_solv_*), Coulombic energy (*ΔG_coul_*), non-electrostatic contributions (*ΔG_non-elec_*), and total binding free energy (*ΔG_b_*). Among the evaluated systems, the 010 plane yielded the most favorable (i.e., most negative) *ΔG_b_* values, driven primarily by strong electrostatic and solvation contributions (Figure 9b). The large negative *ΔG_solv_* values are consistent with the highly charged nature of MSU crystals, which present exposed ionic species-particularly Na+ ions capable of strong interactions with both the solvent and PGAL. In Poisson–Boltzmann-based approaches, solvent polarization plays a dominant role in stabilizing charged systems [35]. Thus, large negative *ΔG_solv_* values are expected and reflect strong electrostatic coupling between the solute and solvent, rather than an anomalous result. This contribution is particularly pronounced for the 010 plane, likely due to increased accessibility of Na+ ions. The *ΔG_coul_* term also contributes substantially to *ΔG_b_*, indicating that electrostatic interactions are the primary driving force for complex formation. Additionally, the MSU 010 system exhibited a higher number of hydrogen bonds and specific interactions with Na^+^ ions, promoting a highly stable binding interface. Coordination of PGAL with multiple Na^+^ ions enhances charge complementarity and leads to strong ion-mediated stabilization. In contrast, the *ΔG_non-elec_* term remained relatively small across all systems, suggesting that van der Waals and hydrophobic interactions play a secondary role. Overall, *ΔG_b_* is dominated by the combined effects of solvation and electrostatic contributions, leading to highly favorable binding energies for the 010 system. However, this behavior likely reflects an idealized scenario in which the crystal structure maximizes ion accessibility and electrostatic interactions.

In contrast, the 011 system represents a more physiologically relevant crystal plane and exhibits significantly less negative contributions of *ΔG_solv_*, *ΔG_coul_* and *ΔG_b_*. The reduced electrostatic contribution suggests limited accessibility of ionic sites and a less optimized interaction interface, more closely reflecting the complexity of in vivo conditions. Despite this, the interaction remains energetically favorable, supporting the formation of PGAL-urate complexes under physiological conditions (Figure 9c).

For the 001 system, calculations were not performed, as molecular docking did not yield stable or meaningful binding poses *ΔG_b_* ng poses (Figure 9a). The absence of favorable interactions suggests that this crystallographic orientation does not provide a suitable interface for PGAL binding, further supporting the structural dependence of the interaction. These findings indicate that idealized crystal models may overestimate binding affinity compared to experimentally derived structures. Therefore, while the 010 plane provides insight into the maximum interaction potential, the 011 system offers a more realistic representation of PGAL-MSU interactions under biological conditions. Overall, the results support an ion-mediated binding mechanism, in which electrostatic complementarity and Na+ ion coordination play a central role, whereas non-electrostatic interactions contribute only marginally to complex stabilization.

### 3.12. Analysis of Electrostatic Potential Maps for the Most Stable Docking Poses

**Figure 10:**
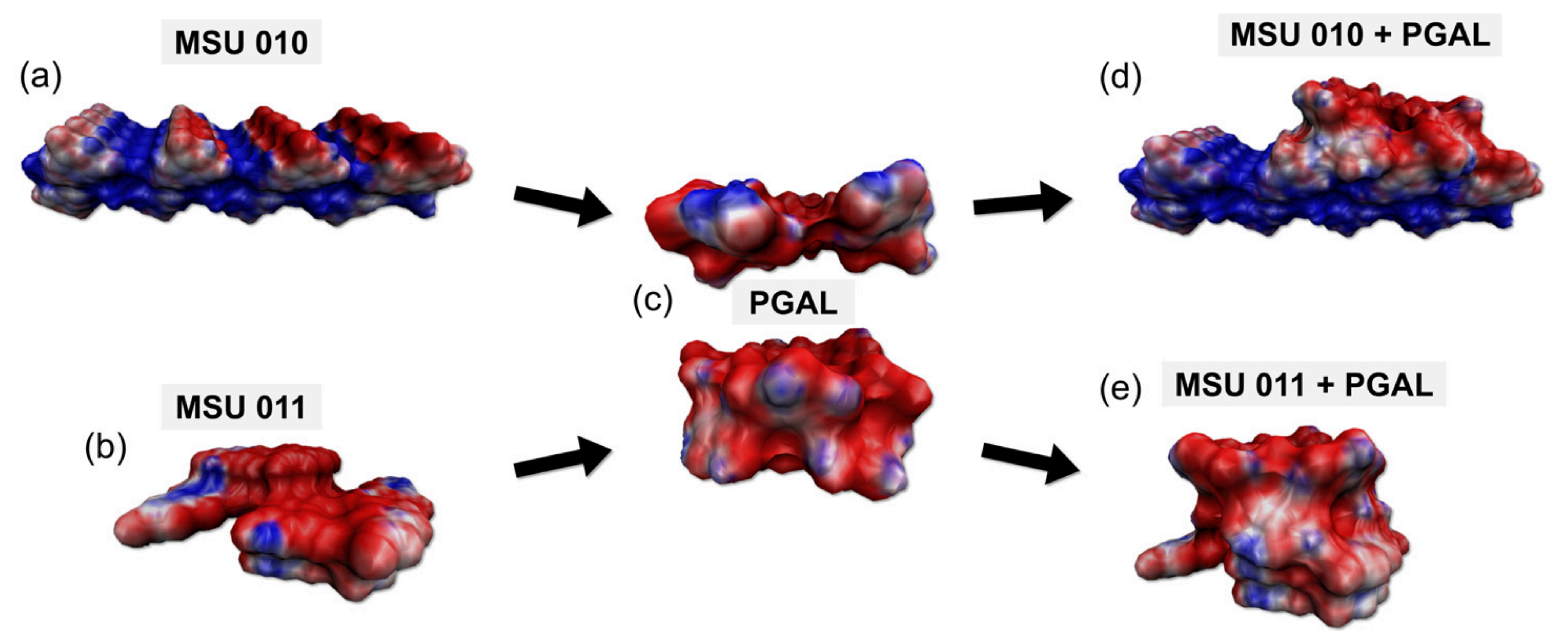
Electrostatic potential of the system. (**a**,**b**) MSU crystals. (**c**) PGAL. (**d**,**e**) PGAL/MSU complexes. According to the APBS color scale, blue indicates positive electrostatic potential, red indicates negative electrostatic potential, and white indicates near-zero electrostatic potential. The visualization was obtained by Visual Molecular Dynamics (VMD).

Electrostatic potential calculations were performed for complex 1, identified as the most stable docking configuration (Figure 10) for both the PGAL-MSU (010) and PGAL-MSU (011) systems based on *ΔG_b_* values. The resulting maps reveal a clear charge complementarity between PGAL, which exhibits a predominantly negative surface, and MSU crystals, where positive regions are primarily associated with Na^+^ ions. This finding supports the dominant role of electrostatic interactions in complex formation. The electrostatic potential maps illustrate the charge distribution in each component: PGAL displays a largely negative potential (Figure 10a), whereas the MSU crystal surface presents localized positive regions corresponding to exposed Na^+^ ions (Figure 10b). This complementary charge distribution promotes attractive interactions between the anionic sites of PGAL and the cationic regions of the surface, facilitating stable complex formation. These observations are consistent with the free energy analysis, in which Coulombic contributions represent the primary force for interaction.

## 4. Discussion

The synthetic MSU crystals reproduced the characteristic needle-shaped morphology and negative birefringence of crystals isolated from the synovial fluid of patients with gout. XRPD analysis further confirmed the monosodium urate monohydrate crystalline phase, supporting the use of the synthetic crystals as a controlled and structurally representative stimulus. Although clinically derived crystals exhibited greater morphological heterogeneity, aggregation, and surface-associated amorphous material, likely reflecting the influence of synovial fluid components [42], the homogeneous synthetic crystals provided a reproducible system for investigating PGAL-MSU interactions. Nevertheless, this model does not fully reproduce the physicochemical complexity of crystals present in the joint environment.

The physicochemical characterization of the PGAL shows the absence of resolved ^1^H NMR resonances attributable to free gallic acid, suggesting that no detectable low-molecular-weight precursor remained under the analytical conditions used. Although the absence of well-defined signals does not in itself constitute proof of polymerization, this result is consistent with the formation of a highly heterogeneous polymeric structure, where the restricted mobility of the chains and the broadening of the signals limit spectral resolution. These results are consistent with SEC analysis, which showed a Mn of 5644 Da and a molecular polydispersity index of 1.946, indicating a relatively broad molecular weight distribution characteristic of polymers obtained through oxidative polymerization processes. The molecular heterogeneity observed by SEC was also consistent with DLS. High PdI values indicate a broad distribution of hydrodynamic sizes in solution, a phenomenon that can be attributed to the coexistence of polymer chains with different lengths and conformations, as well as to the formation of intermolecular associations favored by phenolic hydroxyl and carboxyl groups capable of forming hydrogen bonds and noncovalent interactions in an aqueous medium [43,44]. The SEC and DLS results show that PGAL constitutes a structurally heterogeneous polymeric system at both the molecular and colloidal levels. Zeta potential analysis performed in NaCl solution yielded a value of −1.561 ± 1.71 mV, indicating a slight negative surface charge. This behaviour can be explained by ionizable functional groups derived from gallic acid. As the pH increases, these functional groups undergo progressive deprotonation; initially, the carboxyl group (pKa ≈ 4) is deprotonated, while the phenolic hydroxyl groups require higher pH values to ionize [45,46]. Under physiological conditions, most of PGAL’s negative charge can be attributed to the carboxylate groups (–COO^−^), while the phenolic groups remain largely protonated. Consequently, PGAL behaves as an anionic polyelectrolyte, whose surface charge depends on the degree of ionization of its functional groups.

PMA-induced differentiation established a macrophage-like THP-1 model suitable for evaluating crystal-induced responses. Differentiated cells exhibited the expected adherent morphology and a significant reduction in HLA-DR expression. These phenotypic changes, together with the enhanced IL-1β response to MSU stimulation, supported the functional responsiveness of the model. However, because CD44 and HLA-DR represent a limited phenotypic panel, these findings should be interpreted as validation of macrophage-like differentiation [47,48]. Functionally, PMA stimulation showed increased basal IL-1β production, consistent with the primed inflammatory state described in THP-1-derived macrophage models [49]. Subsequent MSU exposure further increased IL-1β secretion, supporting the responsiveness of the model to crystal stimulation [50,51].

MSU crystal exposure induced morphological alterations consistent with cellular stress, including cytoplasmic retraction and partial loss of adhesion, in agreement with previous reports of crystal-induced cellular damage [52,53]. PGAL showed no evidence of basal cytotoxicity and attenuated the morphological alterations observed under MSU crystal stimulation [23]. Although crystal violet staining showed no significant differences in adherent cell density among experimental groups, the MTT assay revealed reduced metabolic activity following MSU exposure that was significantly increased by PGAL treatment. These differences likely reflect the distinct biological endpoints assessed by each assay, as crystal violet primarily estimates adherent cell density, whereas MTT reflects cellular metabolic activity [28]. Annexin V/EthD-1 analysis indicated that MSU exposure was associated with a numerical reduction in cell viability and an increase in apoptotic cells, increasing the population of cells displaying both phosphatidylserine externalization and loss of plasma membrane integrity. These alterations are consistent with the cellular stress induced by crystals, which has been linked to mitochondrial dysfunction, oxidative stress, lysosomal damage, and activation of regulated cell-death pathways [14,53]. Although PGAL treatment was associated with numerical improvements in cell viability and reductions in early apoptotic cells, these changes did not reach statistical significance. Both PGAL concentrations significantly reduced the proportion of cells exhibiting combined phosphatidylserine externalization and membrane damage, suggesting attenuation of MSU-induced cellular injury. Nevertheless, additional studies using pathway-specific markers are required to clarify the precise mechanisms by which PGAL modulates cell-death pathways.

Phagocytosis of MSU crystals is an early event in gout pathophysiology, as crystal internalization and subsequent phagolysosomal processing contribute to oxidative stress and inflammasome signaling [54,55,56]. In this study, PGAL markedly reduced the pHrodo positive signal, indicating interference with acidification-dependent phagocytic activity. However, this assay does not distinguish between reduced particle uptake and altered post-internalization acidification. Complementarily, polarized light microscopy demonstrated reduced MSU crystal internalization following PGAL treatment, supporting modulation of early crystal–macrophage interactions. To determine whether this effect was accompanied by changes in the soluble urate fraction, urate concentrations were quantified in the culture medium. Although numerically lower soluble urate levels were observed following PGAL treatment, these differences were not statistically significant, providing no evidence of an effect on soluble urate. Nevertheless, the reduced MSU crystal internalization observed with PGAL was accompanied by a significant decrease in MSU-induced NLRP3 protein expression at both PGAL concentrations. IL-6 and IL-1β levels were also significantly reduced by P1 compared with the MSU group. These findings suggest that modulation of early crystal-macrophage interactions by PGAL is associated with attenuation of downstream inflammatory responses, particularly NLRP3 expression and IL-1β production, consistent with the anti-inflammatory effects reported for certain polyphenols in experimental models of gout [57,58,59].

Consistent with the pro-oxidative environment associated with MSU-induced inflammation, MSU crystal exposure increased intracellular ROS and significantly elevated the O_2_^•^-associated signal, while NO-associated fluorescence was numerically higher but did not differ significantly from the control. In this context, PGAL significantly reduced intracellular ROS in MSU-stimulated cells, whereas the numerically lower O_2_^•^ signal did not reach statistical significance compared with the MSU-group. These findings suggest that the antioxidant effects of PGAL may not involve a direct reduction in O_2_^•^ generation under the evaluated conditions, although further studies are required to identify the predominant cellular sources of ROS and the potential involvement of enzymatic systems. [60,61]. PGAL also significantly reduced NO-associated fluorescence in MSU-stimulated cells without affecting basal levels. Together, the reductions in ROS and NO indicate attenuation of nitro-oxidative stress, which may contribute to the observed decrease in NLRP3 inflammasome activation and IL-1β secretion [62,63,64].

Zeta-potential analysis provided complementary information on the electrokinetic behavior of PGAL and MSU crystals. Both PGAL concentrations exhibited negative zeta-potential values, with a shift toward a more negative potential at the higher PGAL concentration, consistent with the anionic characteristic of the polymer [65]. When PGAL was combined with MSU crystals, the mixed dispersions retained zeta-potential values close to those of MSU crystals alone, indicating that their overall electrokinetic behavior was predominantly associated with the crystal-containing phase. A similar pattern was observed in the series incubated under controlled conditions. While PGAL exhibited more negative zeta-potential values, both PGAL–MSU systems remained electrokinetically close to MSU crystals. The behavior observed indicates that PGAL did not substantially alter the overall zeta potential of the crystal-containing dispersions. This finding does not exclude localized polymer association with the crystal surface, since such interactions may occur without producing a substantial change in the overall electrokinetic potential measured at the slipping plane [66]. From a biological perspective, such localized surface association could potentially modify the availability or accessibility of crystal-surface features involved in cell recognition [67].

To further explore whether the effect of PGAL was associated with direct interactions with crystals, molecular docking analysis we performed. The results indicate that PGAL interacts with the crystal surface primarily through electrostatic forces, particularly via coordination with Na^+^ ions. This aspect is noteworthy, as most studies on polyphenols have focused on interactions with soluble targets such as proteins including IL-17A, xanthine oxidase, or urate transporters [68,69,70] whereas there are relatively few reports addressing interactions with the solid crystalline phase of urate.

The energy decomposition analysis indicated that the Coulombic component constitutes the main contribution to the binding free energy. Electrostatic potential maps further revealed a clear complementarity between the predominantly negative surface of PGAL and the positively charged regions associated with exposed Na^+^ ions on the MSU crystal surface. Although these values reflect the behavior of a computational model and do not represent direct experimental affinities, the observed pattern supports a mechanism in which PGAL binds to the crystal surface and may modulate key physicochemical properties, such as surface charge and interactions with cellular recognition receptors. Future studies integrating experimental and computational approaches will be necessary to validate these interactions and further elucidate the mechanistic basis for therapeutic strategies aimed at targeting PGAL-MSU crystal interactions. The present study has several limitations that should be acknowledged. Although THP-1-derived macrophages constitute a well-established model for investigating MSU crystal-induced inflammation and NLRP3 inflammasome activation, confirmation of these findings in human synoviocytes would further strengthen their translational relevance, and additional studies integrating both macrophages and synoviocytes would provide a more comprehensive understanding of the cellular mechanisms involved. Finally, the absence of in vivo validation limits the direct extrapolation of these findings to the clinical setting. Future studies using experimental models of crystal-induced inflammation will be necessary to further validate the proposed mechanism of action of PGAL.

## 5. Conclusions

PGAL protected macrophages against MSU crystal-induced inflammatory injury by attenuating oxidative stress and downstream inflammatory responses while reducing crystal phagocytosis. The integration of computational, physicochemical, and biological findings supports a model in which PGAL interacts with the surface of MSU crystals, modifying crystal–cell interactions and thereby limiting their inflammatory potential. Collectively, these findings identify the crystal surface as a promising target for polyphenol-based compounds and provide proof-of-concept evidence for an alternative strategy to modulate crystal-induced inflammation in gout. Further mechanistic, physicochemical, and in vivo studies are warranted to validate this approach.

## Figures and Tables

**Table 1:** XRD parameters of sodium urate monohydrate (MSU) crystals.

Sample	a (Å)	b (Å)	c (Å)	α (°)	β (°)	γ (°)	Ref.
**Reference MSU**	10.888(5)	9.534(3)	3.567(1)	95.06(3)	99.47(5)	97.17(3)	[27]
**Synthetic MSU**	10.8805(4)	9.5148(5)	3.5650(2)	95.119(4)	99.548(4)	97.177(3)	In this work

MSU, monosodium urate crystals.

**Table 2:** Zeta potential of MSU crystals, PGAL and PGAL–MSU mixtures.

Sample	Non-Incubated Aqueous Dispersions (mV)	Dispersions Incubated for 72 h Under Controlled Conditions (mV)
MSU	−31.27 ± 1.04	−20.57 ± 6.55
P1	−24.53 ± 4.81	−34.43 ± 5.00
P2	−31.37 ± 1.45	−40.43 ± 9.95
P1 + MSU	−32.23 ± 1.99	−21.23 ± 6.03
P2 + MSU	−32.17 ± 1.39	−20.60 ± 3.92

**Table 3:** Decomposition of binding free energy contributions for PGAL- MSU 010 and PGAL-MSU 011 complexes.

	ΔG_solv_ (kJ/mol)	ΔG_coul_ (kJ/mol)	ΔG_non-elec_ (kJ/mol)	*ΔG_b_ (kJ/mol)
PGAL- MSU 010				
Complex	−29,597	−4716	−17	−34,330
Complex	−29,341	−4509	−20	−33,870
Complex	−29,413	−4415	−19	−33,847
PGAL- MSU 011				
Complex	−8750	−74	−16	−8840
Complex	−8594	−62	−17	−8673
Complex	−8365	−52	−16	−8433

* $\Delta G_{b}\;=\;\Delta G_{solv}+\;\Delta G_{coul}\;+\;\Delta G_{non-elec}.$

## Data Availability

The original contributions presented in this study are included in the article/Supplementary Material. Further inquiries can be directed to the corresponding author.

## Supplementary Materials

The following supporting information can be downloaded at: https://www.mdpi.com/article/10.3390/polym18151828/s1. Figure S1: X-ray powder diffraction (XRPD) characterization of synthetic and clinical MSU crystals. (a) XRPD patterns of MSU crystals: MSU crystals isolated from human synovial fluid and synthetic MSU crystals. (b). Rietveld refinement of the XRPD pattern of MSU monohydrate, showing the observed pattern (Y_obs_), calculated pattern (Y_calc_), and difference curve (Y_obs_ − Y_calc_), confirming the crystalline phase; Figure S2: ^1^H NMR spectrum of PGAL; Figure S3: Validation of the THP-1-derived macrophage model. (a) Untreated THP-1 cells remained in suspension, (b) PMA-treated cells acquired a macrophage-like phenotype characterized by increased adherence and cellular spreading. (c) CD44 expression and (d) HLA-DR expression in untreated and PMA-treated cells. (e) Basal secretion of IL-1β by untreated and PMA-differentiated THP-1 cells. (f) PMA-differentiated THP-1 macrophages stimulated with increasing concentrations of MSU crystals exhibited enhanced IL-1β production. Data are shown as individual values with mean ± SD. * *p* < 0.05, ** *p* < 0.01; Figure S4: Effect of PGAL on cell viability and cell-death-associated populations in MSU crystal-stimulated THP-1-derived macrophages. (a) Quantitative analysis of An-nexin V^−^/EthD-1^+^, (b) Annexin V^+^/EthD-1^+^, (c) No stained cells: Annexin V^−^/EthD-1^−^ (d) Annexin V^+^/EthD-1^−^ cell populations under the indicated experimental conditions. Data are presented as mean ± SD from independent biological experiments. Statistical differences were evaluated using one-way ANOVA followed by Tukey’s multiple-comparisons test * *p* < 0.05 ** *p* < 0.01. Right panel. Representative bivariate flow cytometry dot plots showing Annexin V and EthD-1 fluorescence and quadrant-based classification of the four cell populations under each experimental condition. The lower-left quadrant represents Annexin V^−^/EthD-1^−^ viable cells, the lower-right quadrant Annexin V^+^/EthD-1^−^ cells, the upper-right quadrant Annexin V^+^/EthD-1^+^ cells, and the upper-left quadrant Annexin V^−^/EthD-1^+^ cells; Figure S5: Effect of PGAL on soluble urate levels. MSU crystals significantly increased soluble urate levels compared to the control. Data are expressed as mean ± SD. Statistical analysis was performed using one-way ANOVA followed by Tukey’s post hoc test (**** *p* < 0.0001).

## Abbreviations

The following abbreviations are used in this manuscript:

MSU	Monohydrated monosodium urate
ROS	Reactive oxygen species
TLRs	Toll-like receptors
NLRP3	Cryopyrin complex or inflammasome
Pro-IL-1β	precursor of interleukin-1β
TNF-α	Tumor necrosis factor alpha
NF-kB	Nuclear factor kappa B
MyD88	Myeloid differentiation primary response 88
PI3K	Phosphatidylinositol 3-kinase
NSAIDs	Nonsteroidal anti-inflammatory drugs
PGAL	Poly gallic acid
PMA	Phorbol 12-myristate 13-acetate
FBS	Fetal bovine serum
PS	penicillin-streptomycin
MTT	3-(4,5-dimethylthiazol-2-yl)-2,5-diphenyltetrazolium bromide
PI	Propidium Iodide
DPBS	Dulbecco’s phosphate-buffered saline
carboxy-H_2_DCFDA	5-(and-6)-carboxy-2′,7′-dichlorodihydrofluorescein diacetate
DCF	Dichlorofluorescein
RFU	Relative fluorescence units
O2^•^	Superoxide
DHE	dihydroethidium
DAF-FM	4-amino-5-methylamino-2′,7′-difluorofluorescein diaceta
CCDC	Cambridge Crystallographic Data Centre
*ΔG_b_*	Binding free energy
*ΔG_elec_*	Electrostatic energy
*ΔG_non-elec_*	Non-electrostatic energy
*ΔG_coul_*	Coulombic energy
*ΔG_solv_*	Solvation energy
APBS	Adaptive Poisson-Boltzmann
ASA	Solvent-accessible surface area
VMD	Visual Molecular Dynamics
R_wp_	Weighted-profile R-factor
