# Supplementary material for: Electrostatic Interaction of Enzyme-Mediated Poly(gallic acid) with Monosodium Urate Crystals Attenuates Inflammasome Activation and Oxidative Stress
Source: Polymers (Basel). 2026 Jul 26;18(15):1828. doi: 10.3390/polym18151828 (PMC13467736; doi:10.3390/polym18151828)
Supplement: Supplementary file 1 [file polymers-18-01828-s001.zip › polymers-4407893-Supplementary Materials.pdf]

Supplementary information

Electrostatic interaction of enzyme-mediated poly(gallic acid) with monosodium urate crystals attenuates inflammasome activation and oxidative stress

Iris N. Serratos[1], Luis Angel Carrasco-Sánchez[2], Karina Martínez-Flores[3], Roberto Sánchez-Sánchez[4], Valentín Martínez-López[4], Ambar López-Macay[3], Jesús Cervantes-Meneses[3], Alberto Carlos-Martínez[5], Rafael Guillermo Suárez-Nájera[6], Alfredo Jiménez-Mondragón[1], Ingrid Salgado-Gutiérrez[4], Carmen Hernández-Valencia[7], Mónica Olvera-Barranco[8], Janitzia- Vázquez-Mellado[9], Israel Alfonso Núñez-Tapia[10], Miquel Gimeno[8], Javier Fernández-Torres[3] and Yessica Zamudio-Cuevas[3,*]

[1]Universidad Autónoma Metropolitana, Departamento de Química. Avenida Ferrocarril San Rafael Atlixco. No. 186. Col. Leyes de Reforma 1ª Sección, Alcaldía Iztapalapa, Ciudad de México. México. C.P. 09310.

[2]Universidad Autónoma Metropolitana, Departamento de Ciencias Biológicas y de la Salud. Avenida Fe-rrocarril San Rafael Atlixco. No. 186. Col. Leyes de Reforma 1ª Sección, Alcaldía Iztapalapa, Ciudad de México. México. C.P. 09310.

[3]Laboratorio de Líquido Sinovial. Instituto Nacional de Rehabilitación Luis Guillermo Ibarra Ibarra, Cal-zada México-Xochimilco. No. 289. Col. Arenal de Guadalupe, Alcaldía Tlalpan, Ciudad de México, México. C.P. 14389.

[4]Unidad de Ingeniería de Tejidos, Terapia Celular y Medicina Regenerativa. Instituto Nacional de Rehabi-litación Luis Guillermo Ibarra Ibarra, Calzada México-Xochimilco. No. 289. Col. Arenal de Guadalupe, Alcaldía Tlalpan, Ciudad de México, México. C.P. 14389.

[5]Unidad de Síntesis de Evidencia. Instituto Nacional de Rehabilitación Luis Guillermo Ibarra Ibarra, Cal-zada México-Xochimilco. No. 289. Col. Arenal de Guadalupe, Alcaldía Tlalpan, Ciudad de México, México. C.P. 14389.

[6]División de Ciencias Básicas, Facultad de Ingeniería, Universidad Nacional Autónoma de México, Cir-cuito exterior S/N, Coyoacán, Ciudad Universitaria, Ciudad de México, México C.P. 04510.

[7]Universidad Autónoma Metropolitana, Departamento de Biotecnología. Avenida Ferrocarril San Rafael Atlixco. No. 186. Col. Leyes de Reforma 1ª Sección, Alcaldía Iztapalapa, Ciudad de México. C.P. 09310.

[8]Facultad de Química, Departamento de Alimentos y Biotecnología, Universidad Nacional Autónoma de México, Circuito exterior S/N, Coyoacán, Cd. Universitaria, Ciudad de México, México. C.P. 04510.

[9]Departamento de Reumatología, Hospital General de México Eduardo Liceaga, Dr. Balmis No. 148 Col. Doctores, Alcaldía Cuauhtémoc, Ciudad de México, México. C.P. 06720.

[10]Laboratorio de Materiales, Instituto de Investigación en Materiales, Universidad Nacional Autónoma de México, Circuito exterior S/N, Coyoacán, Cd. Universitaria, Ciudad de México, México C.P. 04510.

*Correspondence: yesszamudio@gmail.com

Keywords: Poly gallic acid, monosodium urate crystals, oxidative stress, inflammatory, inflammasome, computational modeling.

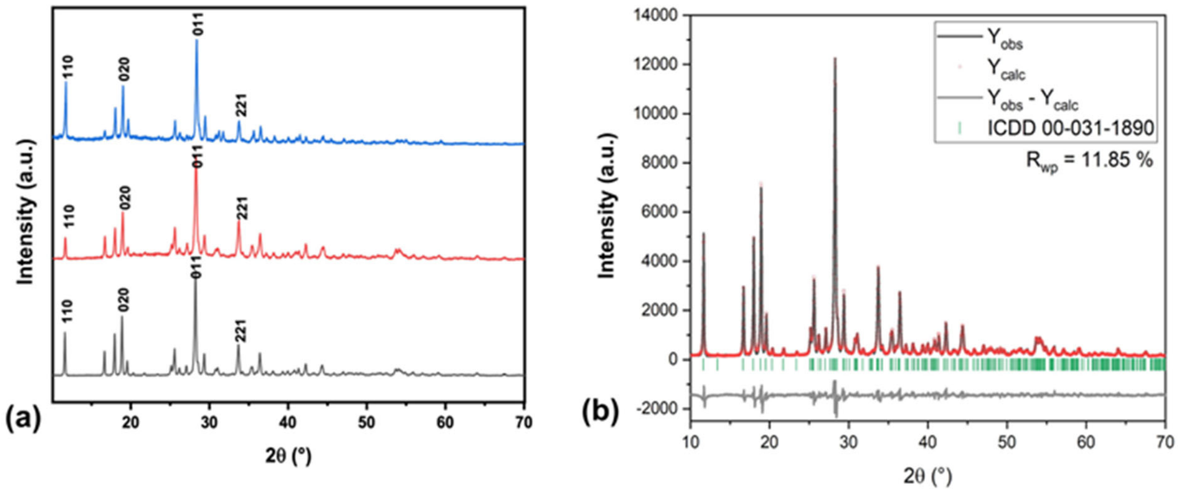

Supplementary Figure S1. X-ray powder diffraction (XRPD) characterization of synthetic and clinical MSU crystals. (**a**) XRPD patterns of MSU crystals: MSU crystals isolated from human synovial fluid and synthetic MSU crystals. (**b**). Rietveld refinement of the XRPD pattern of MSU monohydrate, showing the observed pattern (Y_{obs}), calculated pattern (Y_{calc}), and difference curve ($Y_{obs} - Y_{calc}$), confirming the crystalline phase.

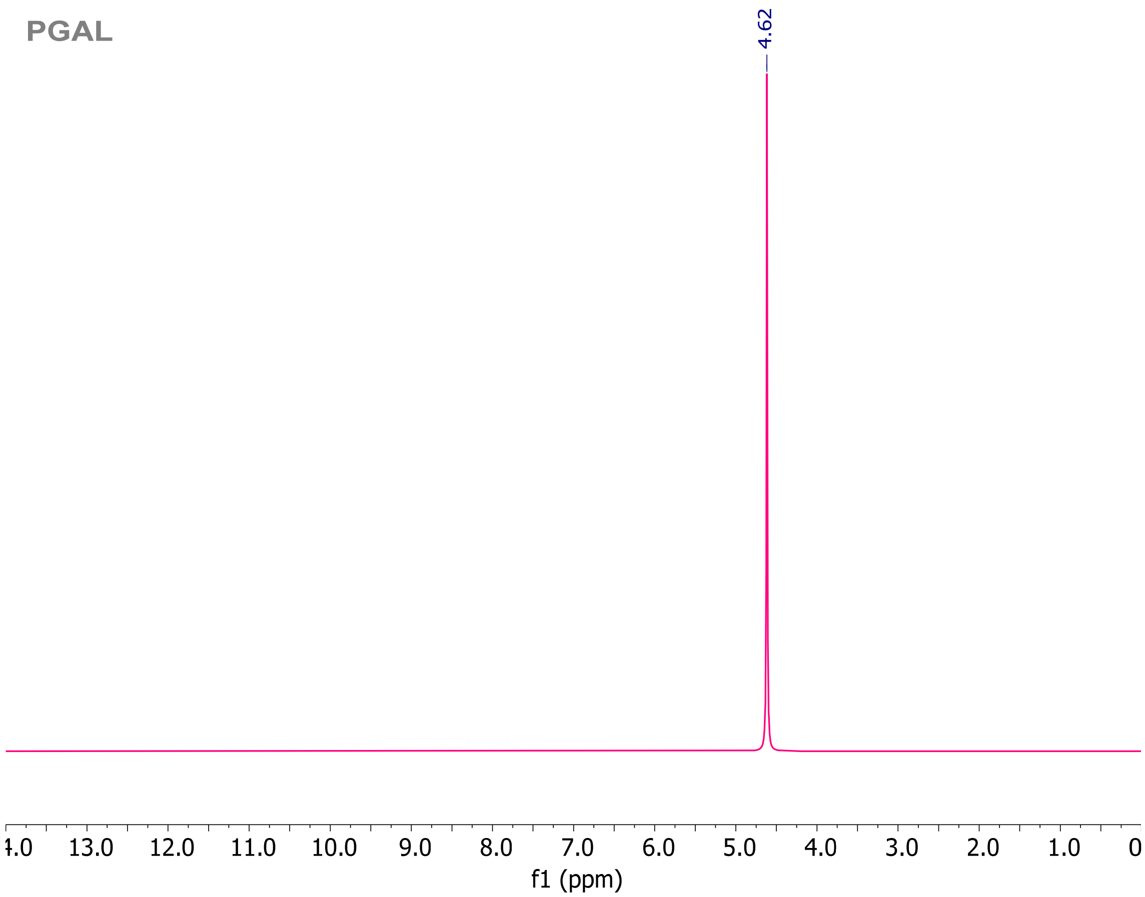

Supplementary Figure S2. ^{1}H NMR spectrum of PGAL

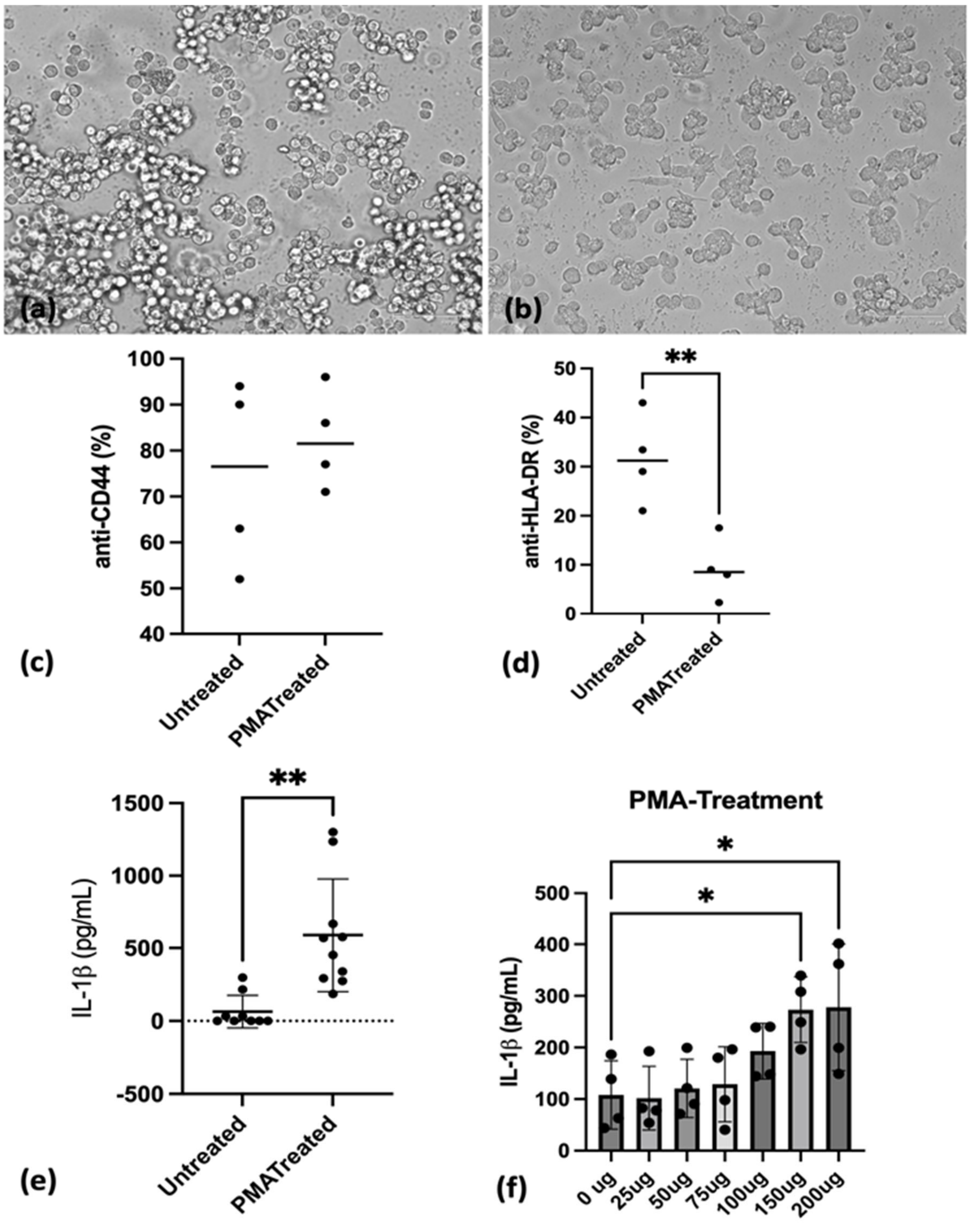

Supplementary Figure S3. **Validation of the THP-1-derived macrophage model.** Validation of the THP-1-derived macrophage model. (a) Untreated THP-1 cells remained in suspension, (b) PMA-treated cells acquired a macrophage-like phenotype characterized by increased adherence and cellular spreading. (c) CD44 expression and (d) HLA-DR expression in untreated and PMA-treated cells. (e) Basal

secretion of IL-1β by untreated and PMA-differentiated THP-1 cells. (f) PMA-differentiated THP-1 macrophages stimulated with increasing concentrations of MSU crystals exhibited enhanced IL-1β production. Data are shown as individual values with mean ± SD. *p < 0.05, **p < 0.01.

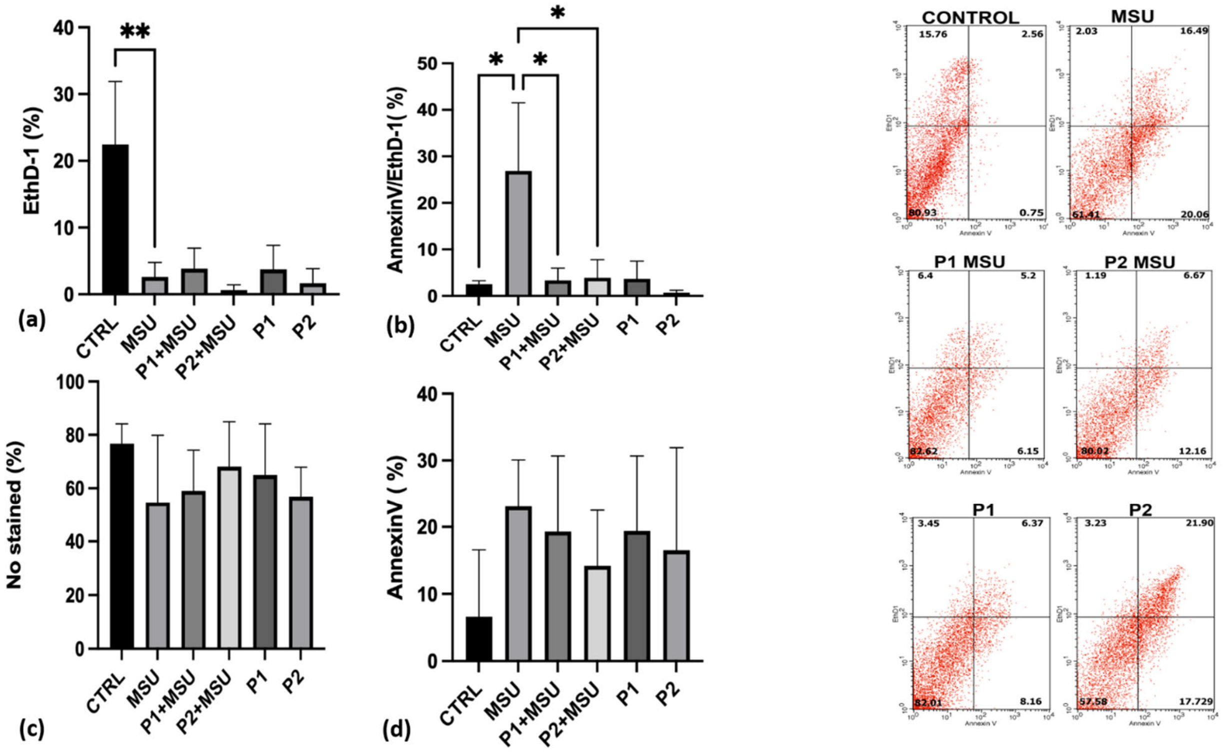

Supplementary Figure S4. **Effect of PGAL on cell viability and cell-death-associated populations in MSU crystal-stimulated THP-1-derived macrophages.** (a) Quantitative analysis of Annexin V⁻/EthD-1⁺, (b) Annexin V⁺/EthD-1⁺, (c) No stained cells: Annexin V⁻/EthD-1⁻ (d) Annexin V⁺/EthD-1⁻ cell populations under the indicated experimental conditions. Data are presented as mean ± SD from independent biological experiments. Statistical differences were evaluated using one-way ANOVA followed by Tukey's multiple-comparisons test *p < 0.05 **p < 0.01. Right panel. Representative bivariate flow cytometry dot plots showing Annexin V and EthD-1 fluorescence and quadrant-based classification of the four cell populations under each experimental condition. The lower-left quadrant represents Annexin V⁻/EthD-1⁻ viable cells, the lower-right quadrant Annexin V⁺/EthD-1⁻ cells, the upper-right quadrant Annexin V⁺/EthD-1⁺ cells, and the upper-left quadrant Annexin V⁻/EthD-1⁺ cells.

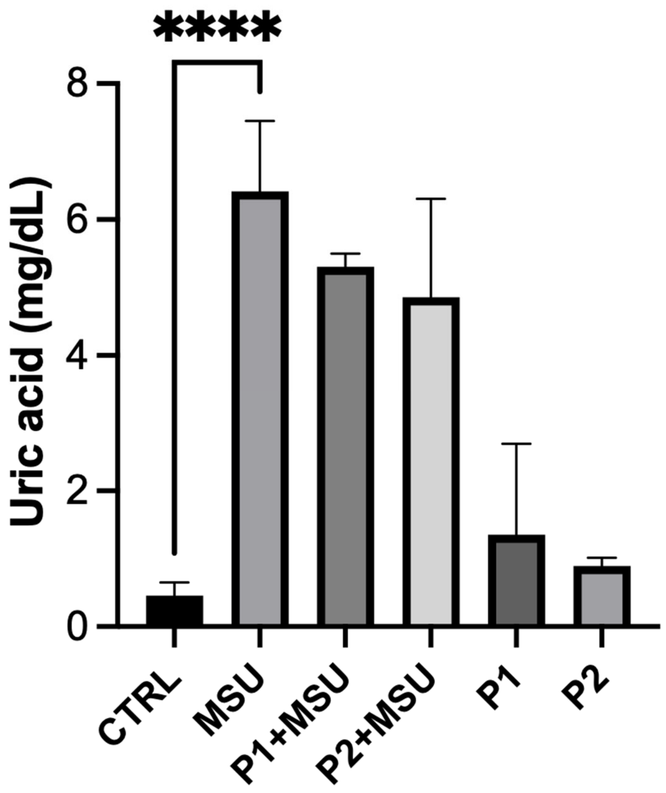

Supplementary Figure S5. Effect of PGAL on soluble urate levels. MSU crystals significantly increased soluble urate levels compared to the control. Data are expressed as mean ± SD. Statistical analysis was performed using one-way ANOVA followed by Tukey's post hoc test (**** $p < 0.0001$).
